# Amphiphilic Cell-Penetrating Peptides Containing Arginine and Hydrophobic Residues as Protein Delivery Agents

**DOI:** 10.3390/ph16030469

**Published:** 2023-03-22

**Authors:** Jonathan Moreno, Khalid Zoghebi, David Salehi, Lois Kim, Sorour Khayyatnejad Shoushtari, Rakesh K. Tiwari, Keykavous Parang

**Affiliations:** 1 Center for Targeted Drug Delivery, Department of Biomedical and Pharmaceutical Sciences, Chapman University School of Pharmacy, Harry and Diane Rinker Health Science Campus, Irvine, CA 92618, USA; 2 Department of Pharmaceutical Chemistry, College of Pharmacy, Jazan University, Jazan 82826, Saudi Arabia

**Keywords:** amphiphilic, cyclic peptides, intracellular transportation, protein delivery

## Abstract

The entry of proteins through the cell membrane is challenging, thus limiting their use as potential therapeutics. Seven cell-penetrating peptides, designed in our laboratory, were evaluated for the delivery of proteins. Fmoc solid-phase peptide synthesis was utilized for the synthesis of seven cyclic or hybrid cyclic–linear amphiphilic peptides composed of hydrophobic (tryptophan (W) or 3,3-diphenylalanine (Dip) and positively-charged arginine (R) residues, such as [WR]_4_, [WR]_9_, [WWRR]_4_, [WWRR]_5_, [(RW)_5_K](RW)_5_, [R_5_K]W_7_, and [DipR]_5_. Confocal microscopy was used to screen the peptides as a protein delivery system of model cargo proteins, green and red fluorescein proteins (GFP and RFP). Based on the confocal microscopy results, [WR]_9_ and [DipR]_5_ were found to be more efficient among all the peptides and were selected for further studies. [WR]_9_ (1–10 µM) + protein (GFP and RFP) physical mixture did not show high cytotoxicity (>90% viability) in triple-negative breast cancer cells (MDA-MB-231) after 24 h, while [DipR]_5_ (1–10 µM) physical mixture with GFP exhibited more than 81% cell viability. Confocal microscopy images revealed internalization of GFP and RFP in MDA-MB-231 cells using [WR]_9_ (2–10 μM) and [DipR]_5_ (1–10 µM). Fluorescence-activated cell sorting (FACS) analysis indicated that the cellular uptake of GFP was concentration-dependent in the presence of [WR]_9_ in MDA-MB-231 cells after 3 h of incubation at 37 °C. The concentration-dependent uptake of GFP and RFP was also observed in the presence of [DipR_5_] in SK-OV-3 and MDA-MB-231 cells after 3 h of incubation at 37 °C. FACS analysis indicated that the cellular uptake of GFP in the presence of [WR]_9_ was partially decreased by methyl-β-cyclodextrin and nystatin as endocytosis inhibitors after 3 h of incubation in MDA-MB-231 cells, whereas nystatin and chlorpromazine as endocytosis inhibitors slightly reduced the uptake of GFP in the presence of [DipR]_5_ after 3 h of incubation in MDA-MB-231. [WR]_9_ was able to deliver therapeutically relevant proteins (Histone H2A) at different concentrations. These results provide insight into the use of amphiphilic cyclic peptides in the delivery of protein-related therapeutics.

## 1. Introduction

Proteins are macromolecules with diverse roles as enzymes, receptors, or channels in cell membranes, catalyzing biochemical reactions and therapeutic agents [1,2,3]. There are about 19,000–20,000 different protein-coding genes in the human genome [4,5]. If we consider the post-translational modification of proteins, such as phosphorylation, cleavage, glycosylation, and acylation, a large number of functionally distinct proteins are present in the human proteome. If viewed from a therapeutics perspective, these estimates represent a huge opportunity to develop protein therapeutics to ease many diseases.

Protein therapeutics hold tremendous promise for curing a variety of illnesses. Many human disorders are caused by the malfunction or dysregulation of a specific protein, and introducing an intact protein to the diseased cell is an alternative to nucleotide-based therapies. Compared to small molecule drugs, protein therapy has several advantages. First, proteins have specificity and complexity in performing a set of functions that cannot be performed by a simple small chemical compound [6,7]. Second, since most proteins have a specific function, it has less effect on normal biological processes, thus, less potential to cause adverse events [8,9]. Third, proteins are endogenous structures that are produced naturally in our bodies. Hence, they are well tolerated and are less likely to elicit immune responses [10,11,12]. Fourth, gene therapy is not currently available for most genetic disorders because of the challenges of nucleic acid delivery. Thus, proteins could be delivered as an alternative option [13]. Lastly, development and acquiring FDA approval have sped up for protein therapeutics [14].

Pharmacokinetic features, such as the route of administration, solubility, stability, and systemic distribution, can limit the successful application of therapeutic proteins [15,16,17,18]. Furthermore, therapeutic proteins are susceptible to systemic cleavage via protein-modifying chemicals and proteases [19,20,21]. To act intracellularly, therapeutic proteins must bypass the plasma membrane. Unlike low molecular weight therapeutics, proteins are large molecules in nature with hydrophilic and hydrophobic properties. These characteristics make the entry of proteins through the cell membrane very challenging, thus restricting their use as potential therapeutics.

Cell-penetrating peptides (CPPs) have been used for the intracellular delivery of a wide range of molecules, including large proteins [22,23]. CPPs are typically composed of 5–40 amino acids, possessing a net positive charge at physiological pH due to several positively charged residues, such as arginine and lysine, that interact with negatively charged phospholipids and promote translocation [24].

Generally, cyclic CPPs have different uptake mechanisms depending on the physicochemical properties, the primary and secondary structure, concentration, membrane structure and type of cells, incubation time, and cargo type [25]. Two main mechanisms of permeation through cell membranes have been proposed in the literature: direct membrane translocation via energy-independent pathways and endocytosis pathways, which require energy consumption [26]. Direct translocation occurs due to electrostatic interactions between positively charged residues of CPPs and negatively charged phospholipid bilayer and is further classified into different models, such as the carpet model, pore formation, and the inverted micelle model [27,28,29]. Conversely, endocytosis is an energy-dependent and active mechanism composed of various pathways, including phagocytosis and pinocytosis, which are classified into macropinocytosis, clathrin-dependent endocytosis, caveolin-dependent endocytosis, and clathrin- and/or caveolin-independent endocytosis [30,31,32].

Hundreds of peptides have been evaluated for their ability to deliver not only fluorophores but also to deliver macromolecules. Among those, the four famous CPPs (Penetratin, R8, TAT, Transportan) and their cyclic counterparts have been reported as shuttles for the delivery of Green Fluorescent Protein (GFP) and measured the intracellular uptake into the cytosol (fusion method) [33]. The reported results revealed that cyclic cationic peptides were the most efficient transporters among all tested carriers. Another report was about covalently attached cyclic [TAT]-GFP, showing immediate cytosolic and nuclear availability [34]. Their findings revealed that the cyclic-CPP–GFP conjugates were internalized into cells with rapid bioavailability in the cytosol and the nucleus, whereas linear CPP analogs did not confer GFP internalization [34]. Tanaka et al. (2021) evaluated polyhistidine (PHP) peptides for the delivery of proteins into plant cells through covalent conjugation [35]. In another report, Schneider et al. (2019) applied R10 to deliver mCherry protein using a covalent strategy [36].

**Figure 1:**
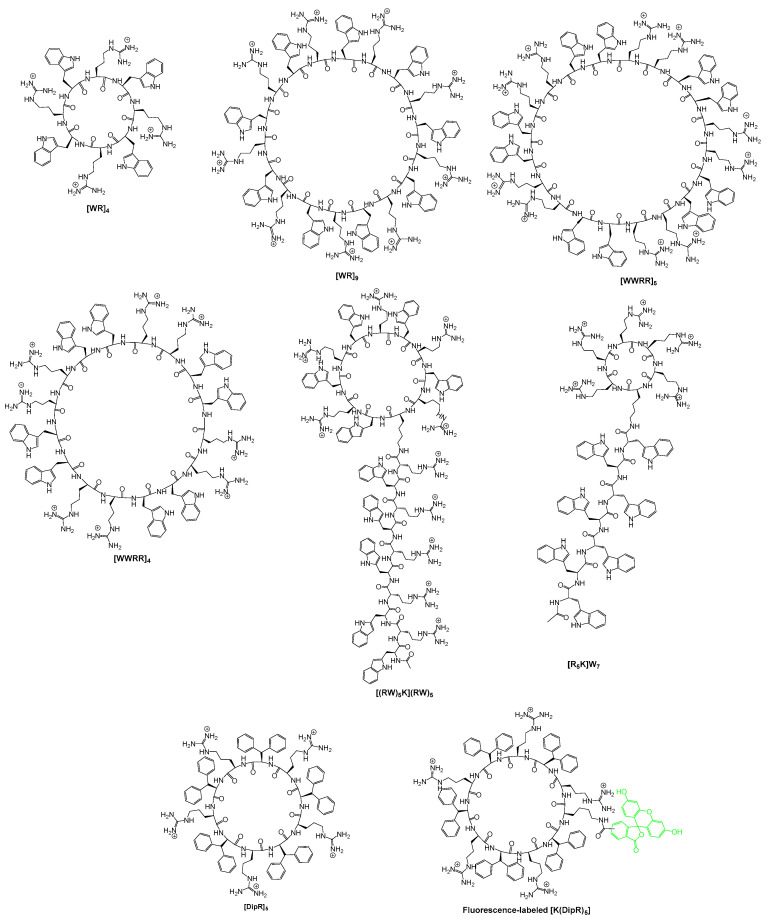
Chemical structures of peptides containing arginine and hydrophobic residues.

We have previously reported that cyclic peptides containing tryptophan (W) and arginine (R) residues, [WR]_4_ [37], [WR]_5_ [38], [WR]_6_, [WR]_7_, [WR]_8_, and [WR]_9_ [39], can act as molecular transporters. The physical mixture of [WR]_9_ (Figure 1) (10 μM) and F′-GpYEEI at (2 μM) significantly enhanced the cellular uptake by 20-fold when compared to F′-GpYEEI alone at the same concentration after 3 h of incubation in CCRF-CEM cells, while the physical mixture of [WR]_5_ (10 μM) with F′-GpYEEI (2 μM) enhanced the uptake by only 4-fold [39]. These results indicate the possibility of using large CPPs as molecular transporters for macromolecules. In fact, many studies have reported multiple efficient CPPs for macromolecule delivery, sharing the feature of the presence of arginine in the sequence [40,41,42,43,44]. In addition to [WR]_4_ [37], [WR]_5_ [38], and [WR]_9_ [39], we have also reported a number of cyclic peptides and hybrid cyclic–linear peptides, [WWRR]_4_ and [WWRR]_5_ [45], [(RW)_5_K](RW)_5_ [46], [R_5_K]W_7_ [47,48], and [DipR]_5_ [49] (Figure 1) as molecular transporters of phosphopeptides, nucleic acids, or small molecules.

Herein, we aimed to investigate the potential of cyclic and cyclic–linear CPPs as delivery agents for proteins. This study is distinct from the previous strategies [34,35,36], focusing on the non-covalent delivery of several proteins using various peptide delivery tools containing R and hydrophobic residues. Figure 1 depicts the chemical structures of selected peptides used herein for evaluating the delivery of proteins. We hypothesized that the positively charged R and hydrophobic residues could have electrostatic and hydrophobic interactions with negatively charged phosphate in the phospholipid bilayer and with charged/hydrophobic residues on the protein leading to more internalization. Different protein models, GFP, red fluorescent protein (RFP), and a clinically relevant protein (Histone H2A) were utilized to assess the ability of peptides to deliver those macromolecules intracellularly. This study expands our knowledge about the ability of cyclic CPPs to efficiently transport functional proteins into cells.

## 2. Results and Discussions

### 2.1. Chemistry

Peptides were synthesized as described previously by us [38,39,45,46,47,48,49]. Fmoc solid-phase peptide synthesis followed by solution-phase cyclization was utilized to synthesize cyclic peptides [39]. In brief, for the synthesis of cyclic peptides, Fmoc-Arg(Pbf)-OH and Fmoc-Trp(Boc)-OH, and Fmoc-L,-3,3-diphenylalanine were used as building block amino acids in peptide synthesis. After the final coupling, the side chain-protected peptide was cleaved from trityl resin by using a cleavage cocktail containing dichloromethane, trifluoroethanol, acetic acid (DCM:TFE:AcOH, 7:2:1 *v*/*v*/*v*, 50 mL). The compounds were directly used for the cyclization reaction. The molecular weights of pure cyclic peptides were confirmed with MALDI-TOF. The peptides were purified using reversed-phase HPLC and lyophilized.

### 2.2. Cytotoxicity of Peptide-Protein Physical Mixture

**Figure 2:**
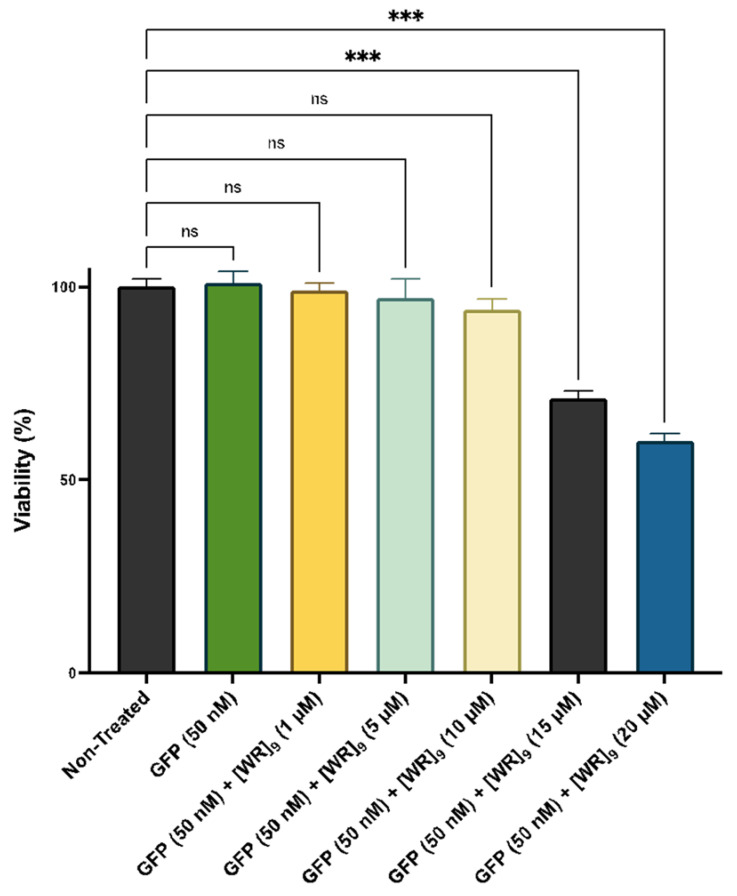
Cytotoxicity of [WR]_9_ with GFP (50 nM) physical mixtures at different peptide concentrations (1–20 µM) in breast cancer (MDA-MB-231) cell line after 24 h. The results are mean ± SD (*n* = 3) (ns; no significance, *** *p* < 0.001 treatments vs. Ctrl (NT).

**Figure 3:**
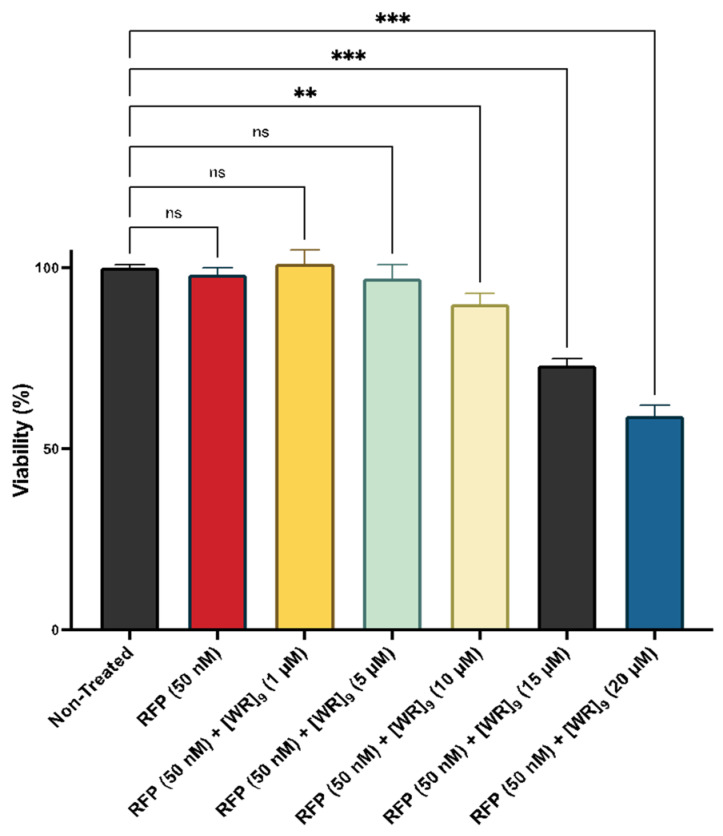
Cytotoxicity of [WR]_9_ with RFP (50 nM) physical mixtures at different peptide concentrations (1–20 µM) in breast cancer (MDA-MB-231) cell line after 24 h. The results are mean ± SD (*n* = 3) (ns; no significance, ** *p* < 0.01, *** *p* < 0.001 treatments vs. Ctrl (NT).

**Figure 4:**
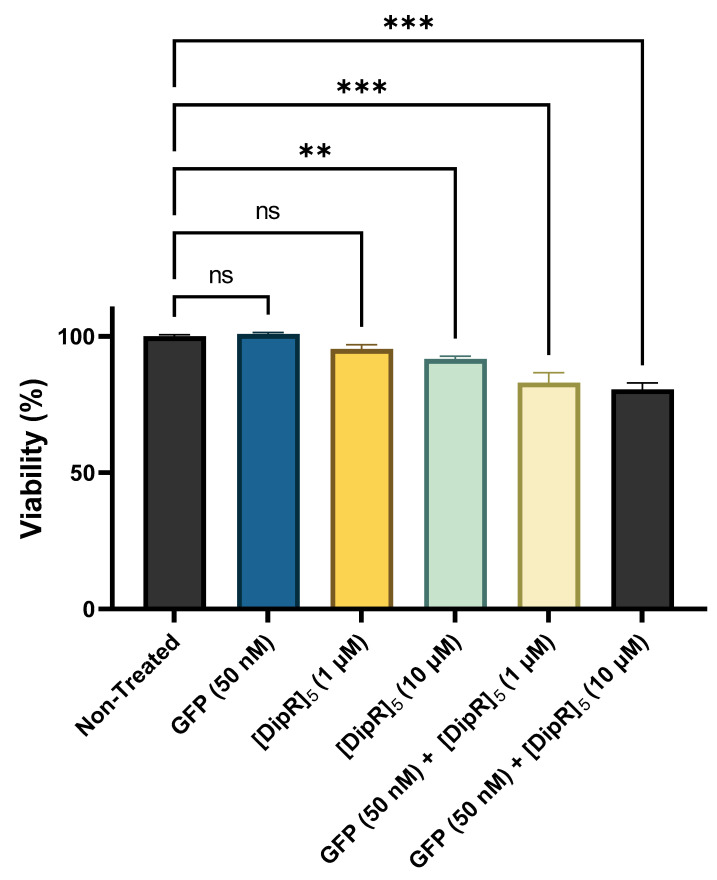
Cytotoxicity of [DipR]_5_ with GFP (50 nM) physical mixtures at different peptide concentrations (1–10 µM) in breast cancer (MDA-MB-231) cell line after 3 h. The results are mean ± SD (*n* = 3) (ns; no significance, ** *p* < 0.01, *** *p* < 0.001 treatments vs. Ctrl (NT).

Cytotoxicity of the peptides has been previously reported by us [38,39,45,46,47,48,49]. For example, [(RW)_5_K](RW)_5_ did not exhibit any significant cytotoxicity in different cell lines, such as human epithelial mammary gland adenocarcinoma cells (MDA-MB-231), human leukemia carcinoma cell line (CCRF-CEM), human ovarian adenocarcinoma cells (SK-OV-3), and human epithelial embryonic kidney healthy (HEK-293) at the concentration of 10 μM after 3 h of incubation [46]. As a representative example, the cytotoxicity of [WR]_9_ with proteins was examined at different concentrations. Fluorescently labeled proteins, such as GFP, RFP, and Cyan Fluorescent Protein (CFP), are used as cellular tags to visualize and track intracellular functions. However, numerous reports suggest that those tags exhibit cellular damage by different mechanisms, including apoptosis initiation, direct damage by reactive oxygen species (ROS) generation, and immunogenicity damage [50,51]. Therefore, to exclude the effect of possible toxicity on the interpretation of experimental data, [WR]_9_-protein (GFP/RFP) mixtures were evaluated for their toxic effect on MDA-MB-231 for 24 h. [WR]_9_ and GFP or RFP were mixed and incubated together for 30 min before adding the cells at room temperature. The GFP/RFP concentration was (50 nM) during the experiment, and the peptide concentration ranged from (1 to 20 µM). The results showed concentration-dependent cytotoxicity. This cytotoxicity is presumably due to higher peptide concentrations in the mixture (15 and 20 µM). GFP alone did not exhibit any sign of cytotoxicity at 50 nM concentration (Figure 2, Table S1 (Supporting Information)). Furthermore, RFP exhibited a similar pattern by revealing concentration-dependent cytotoxicity due to higher [WR]_9_ concentrations, while RFP alone was not cytotoxic at 50 nM (Figure 3, Table S2 (Supporting Information)). The cytotoxicity of cyclic peptides [WWRR]_4_ and [WWRR]_5_ with GFP (50 nM) is shown in Figure S1 and Table S3 (Supporting Information). Additional cytotoxicity studies found no cytotoxicity associated with the increase in GFP concentration alone (100 nM to 500 nM) and in a physical mixture with [WR]_9_ (3 µM) (Figure S2, Table S4 (Supporting Information)). The combination of [DipR]_5_ (Figure 4 and Figure S3, Tables S3 and S5, Supporting Information) with GFP (50 nM to 500 nM) after 3 h in MDA-MB-231 cells showed cell viability of more than 81%. Based on these findings and previous data about the cytotoxicity of other peptides [38,39,45,46,47,48,49] used in this study, cellular uptake experiments were conducted with peptides and protein models (GFP and RFP) concentrations not higher than 10 µM and 50 nM, respectively, and 3 h of incubation.

### 2.3. Cellular Internalization Using Confocal Microscopy

Our goal was to explore the ability of selected CPPs containing hydrophobic residues (W or Dip) and positively-charged arginine (R) residues to direct protein cargoes to localize within the cells, which was monitored using confocal microscopy. As a model protein, we chose GFP and RFP, 27 kDa fluorescent proteins that are commonly used as fluorescence markers in cell microscopy imaging, in a breast cancer cell line (MDA-MB-231). The cells were incubated with the physical mixture of proteins and selected peptides for 3 h in serum-free media, Opti-MEM, to reduce unnecessary interactions between the mixture and transfection media.

**Figure 5:**
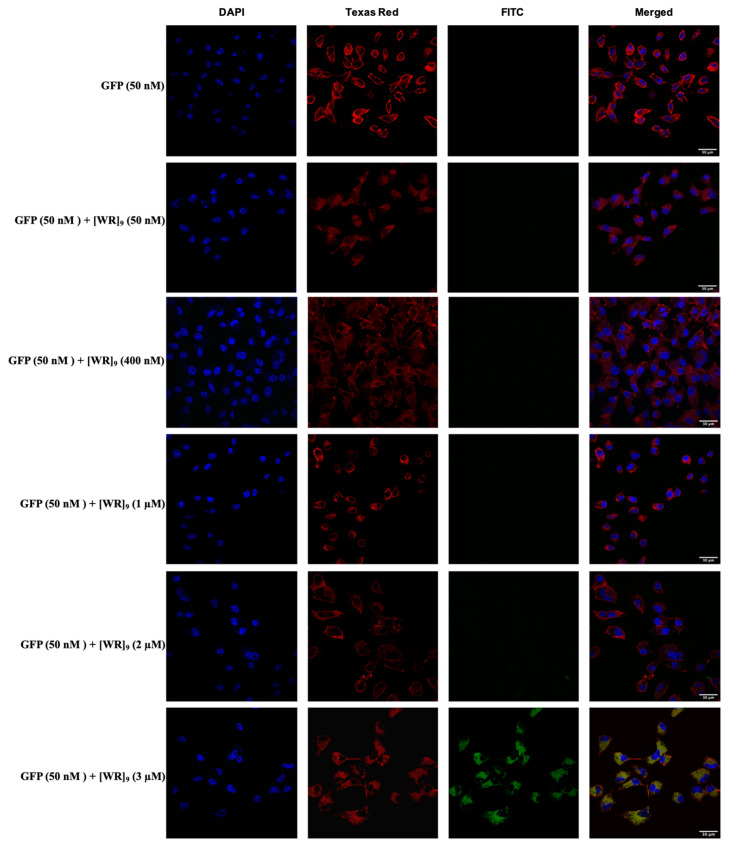
Confocal microscopy images of MDA-MB-231 cells incubated with GFP-[WR]_9_ mixture at a peptide concentration range (50–3 µM) and GFP at (50 nM) for 3 h. The blue, red, and green channels visualize DAPI (used to stain the nucleus), Texas Red (used to stain the cell membrane), and GFP, respectively.

**Figure 6:**
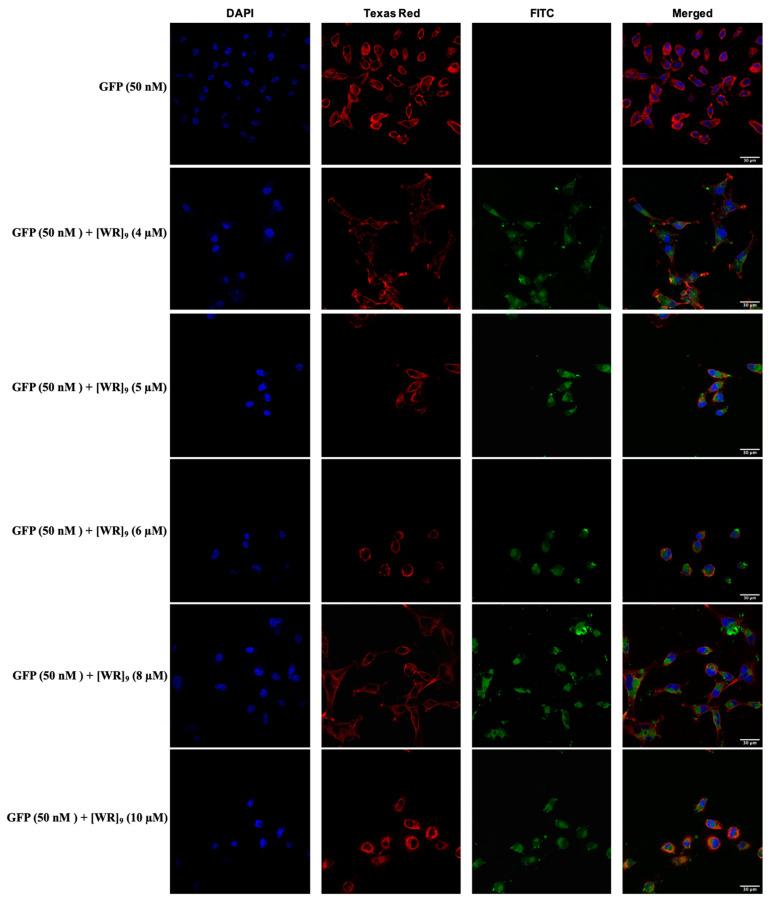
Confocal microscopy images of MDA-MB-231 cells incubated with GFP-[WR]_9_ mixture at a peptide concentration range (4–10 µM) and GFP at (50 nM) for 3 h. The blue, red, and green channels visualize DAPI (used to stain the nucleus), Texas Red (used to stain the cell membrane), and GFP, respectively.

Using GFP, we first studied the optimal time and concentration needed for [WR]_9_ to form proper interactions with the cargo that could lead to internalization into cells. By using (1:1, 1:8, 1:20, 1:40, and 1:60 of GFP:[WR]_9_), we showed that the peptide was able to internalize GFP protein in a concentration-dependent manner (Figure 5). An amount of 50 nM-2 µM of peptide did not show any sign of uptake for GFP inside the cells, and only 3 µM of [WR]_9_ showed cytosolic delivery. Based on this finding, higher [WR]_9_ concentrations (4, 5, 6, 8, and 10 µM) were used with 50 nM GFP in another confocal microscopy experiment to examine the effect of increasing peptide concentrations on GFP cellular internalization. The results showed that at concentrations of 3–10 µM, the uptake of GFP increased intracellularly. However, we noticed cell morphology changed when 10 µM of [WR]_9_ was used, as shown in Figure 6. We have previously shown that [WR]_9_ had minimal toxicity at a concentration of 10 µM in SK-OV-3 and MDA-MB-231 after 24 h of incubation [52]. Herein, as shown in Figure 2 and Figure 3 (Tables S1 and S2), the cell viability was ≥90%. Slight morphological changes are presumably due to the minimal toxicity of this compound at this concentration. Since a significant uptake of the proteins was also observed at concentrations less than 10 µM, this slight morphological change did not affect the uptake of the proteins.

**Figure 7:**
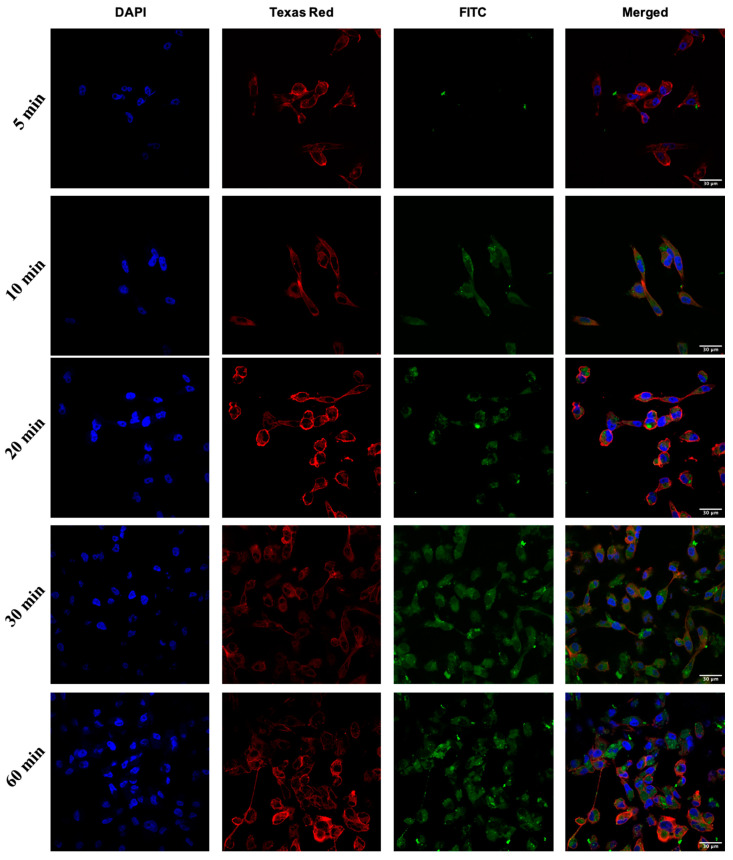
Confocal microscopy images of MDA-MB-231 cells incubated with GFP (50 nM) + [WR]_9_ (4 µM) mixture at different time points (5–60 min). The blue, red, and green channels visualize DAPI (used to stain the nucleus), Texas Red (used to stain the cell membrane), and GFP, respectively.

We also conducted a time-dependent study to evaluate the time needed for [WR]_9_ to deliver GFP inside cells. The time points selected were (5, 10, 20, 30, and 60 min), and the experiment was performed according to the protocol described above using GFP (50 nM) + [WR]_9_ (4 µM). Indeed, the uptake appeared to be time-dependent, and we were able to detect a low signal for GFP uptake as early as 5 min of incubation with the cells, but the intensity of the GFP signal was at the maximum at 30 and 60 min in the cytosol of MDA-MB-231 cells (Figure 7).

Similar studies were conducted with GFP (50 nM) and other peptides, [WR]_4_, [WWRR]_4_, [WWRR]_5_, [(RW)_5_K](RW)_5_, [R_5_K]W_7_, and [DipR]_5_ in MD-MB-231 and/or SK-OV-3 cells. Peptide concentrations ranged from 1 to 10 µM, depending on the previous cytotoxicity studies of the peptides [34,35,41,42,43,44,45] and the results described above.

The results of the confocal microscopy of [WR]_4_ (Figures S4 and S5, Supporting Information), [R_5_K]W_7_ (Figure S6, Supporting Information) [(RW)_5_K](RW)_5_ (Figure S7, Supporting Information), [WWRR]_4_ (Figure S8, Supporting Information), and [WWRR]_5_ (Figure S9, Supporting Information) were provided in the Supporting Information. [WR]_4_ was found to be more efficient in the delivery of GFP in SK-OV-3 cells (Figure S5, Supporting Information) when compared with MDA-MB-231 cells (Figure S4, Supporting Information), suggesting that the uptake for this peptide was cell-dependent, possibly due to the differential nature of interaction with the cell membrane in cancer cells that have different membrane lipid compositions.

We have previously shown that [WR]_4_ enhanced the cellular uptake of cell-impermeable cargo molecules even in the presence of different endocytosis inhibitors [37], indicating that the mechanism of cellular uptake is not dependent exclusively on endocytosis. For [WR]_4_, the direct membrane translocation mainly occurs via an energy-independent pathway and through the electrostatic interactions of positively charged R residues and negatively charged phospholipid bilayer. Bypassing the entrapment in endosomes has significant clinical applications since the cargo molecules can be available immediately for biological activity without the need to be released from endosomes.

**Figure 8:**
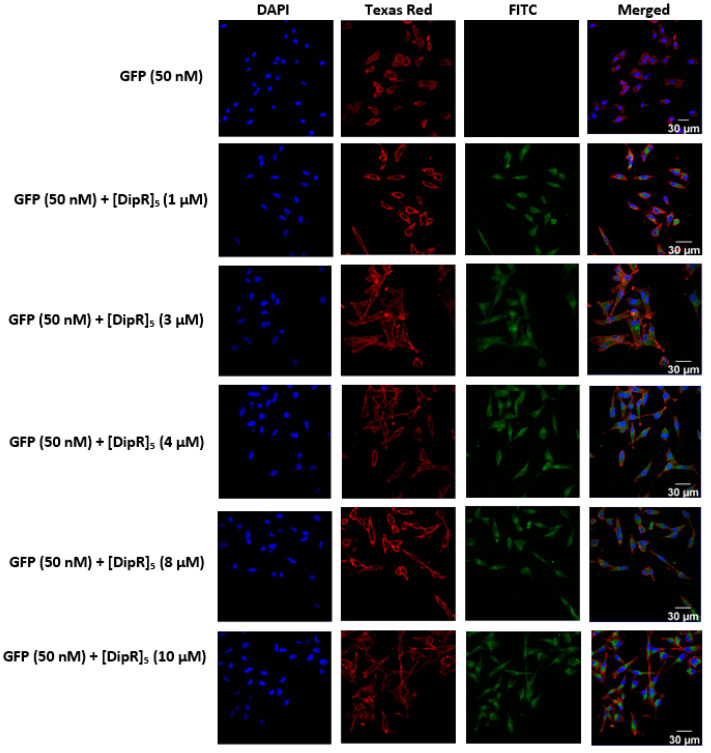
Confocal microscopy images of MDA-MB-231 cells incubated with the GFP-[DipR]_5_ mixture at a peptide concentration range (1–10 µM) and GFP at (50 nM) for 3 h. The blue, red, and green channels visualize DAPI (used to stain the nucleus), Texas Red (used to stain the cell membrane), and GFP, respectively.

**Figure 9:**
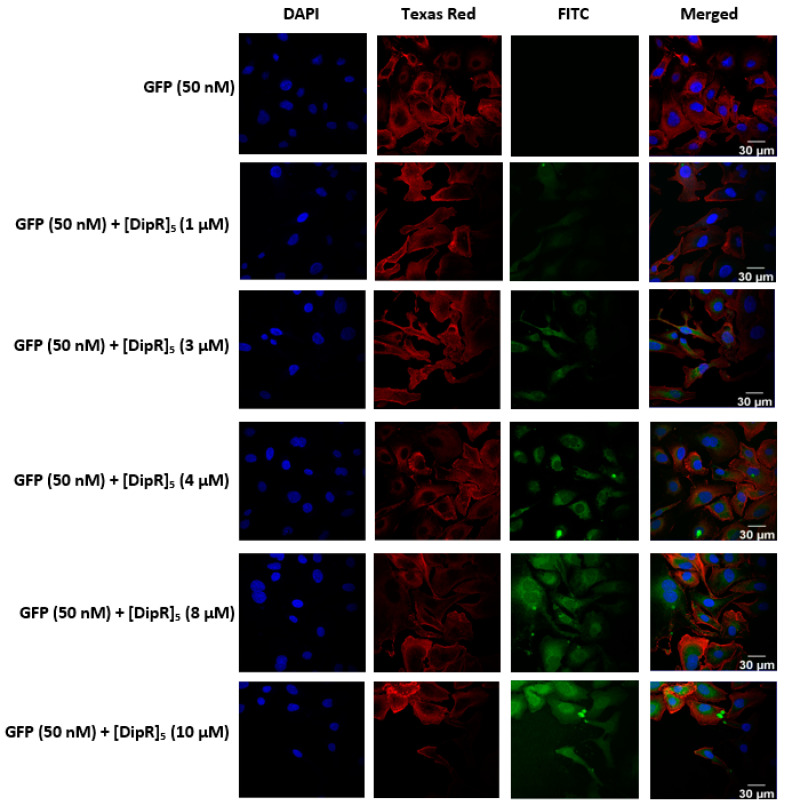
Confocal microscopy images of SK-OV-3 cells incubated with the GFP-[DipR]_5_ mixture at a peptide concentration range (1–10 µM) and GFP at (50 nM) for 3 h. The blue, red, and green channels visualize DAPI (used to stain the nucleus), Texas Red (used to stain the cell membrane), and GFP, respectively.

All peptides were able to deliver GFP to the cells. Among all the peptides, [DipR]_5_ (1–10 µM) (Figure 8 and Figure 9) and [WR]_9_ (3–10 µM) (Figure 5 and Figure 6) showed to be the most efficient peptides in GFP delivery to the cytosol based on the intensity of fluorescence signals, as shown by confocal microscopy. These data suggest that the nature of hydrophobic residues and the number of arginine residues in the peptide significantly affect the GFP delivery since the presence of Dip in [DipR]_5_ and a higher number of R residues in [WR]_9_ versus [WR]_4_ significantly enhanced the GFP uptake.

**Figure 10:**
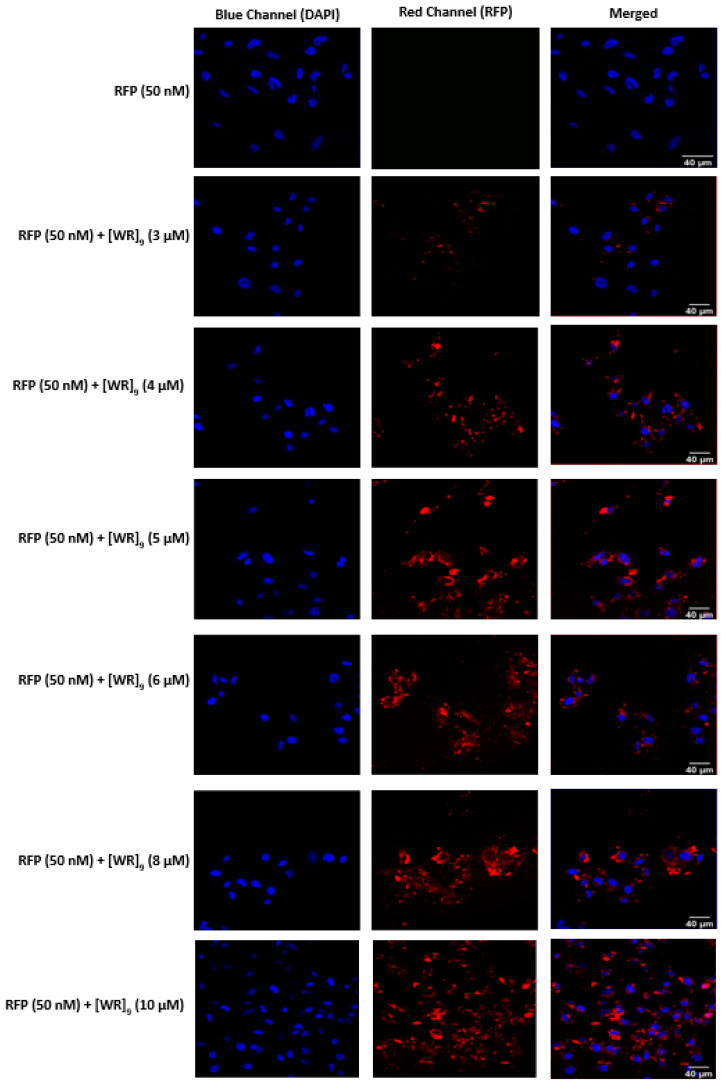
Confocal microscopy images of MDA-MB-231 cells incubated with the RFP-[WR]_9_ mixture at a peptide concentration range (3–10 µM) and RFP at (50 nM) for 3 h. The red and blue channels visualize RFP and DAPI (used to stain the nucleus), respectively.

Based on the confocal microscopy data for efficient delivery of GFP by [WR]_9_ and [DipR]_5_, these two peptides were selected for further studies. Another protein model was also used to confirm the ability of [WR]_9_ to deliver full-length functional protein. Red fluorescence protein (RFP), a 27 kDa fluorescent protein that is commonly used as a fluorescence marker in cell microscopy imaging, was used with the peptide in a breast cancer cell line (MDA-MB-231). Taking advantage of our findings using GFP, cells were incubated with the peptide at concentrations (3–8 µM) and RFP at (50 nM). The results were consistent with GFP confocal images, and all tested peptide concentrations showed cytosolic delivery for RFP. Higher [WR]_9_ concentrations demonstrated increased uptake of RFP (as shown by the red signal of RFP (Figure 10)). GFP and RFP uptake findings proved the ability of cyclic [WR]_9_ as a potential delivery agent for macromolecules with a simple mixing approach and without the need for chemical conjugation or fusion of peptide sequence during protein expression.

**Figure 11:**
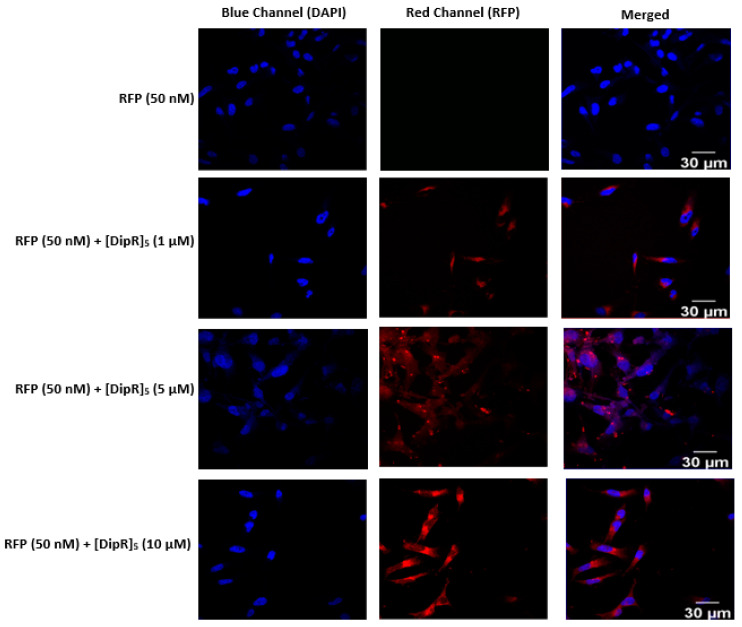
Confocal microscopy images of MDA-MB-231 cells incubated with the RFP-[DipR]_5_ mixture at a peptide concentration (1–10 µM) and RFP at (50 nM) for 3 h. The red and blue channels visualize RFP and DAPI (used to stain the nucleus), respectively.

**Figure 12:**
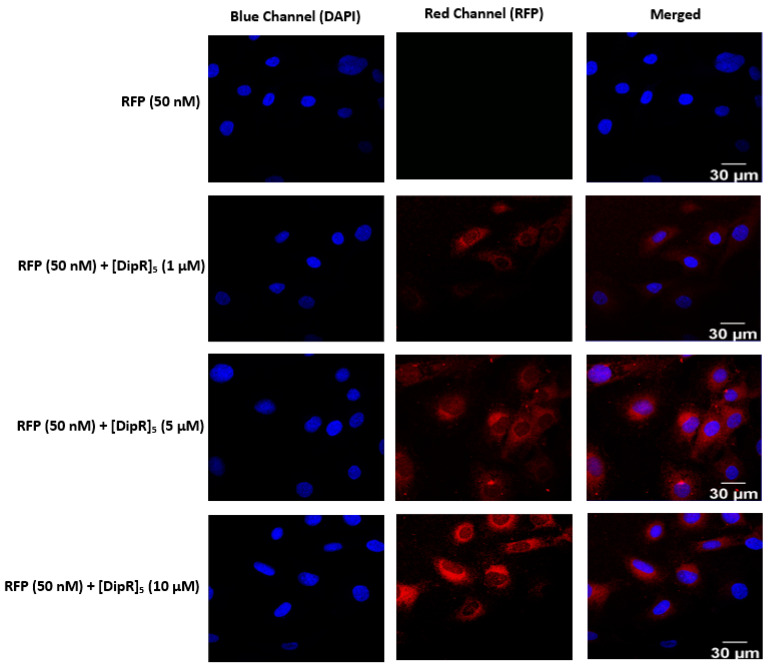
Confocal microscopy images of SK-OV-3 cells incubated with the RFP-[DipR]_5_ mixture at a peptide concentration (1–10 µM) and RFP at (50 nM) for 3 h. The red and blue channels visualize RFP and DAPI, respectively.

As observed for GFP delivery in the presence of [DipR]_5_, this peptide (1–10 µM) was also found to be very efficient in RFP (50 nM) delivery in both MDA-MB-231 and SK-OV-3 cells (Figure 11 and Figure 12). The localization of RFP was found to be in the cytosol, similar to GFP. These data confirm that both [WR]_9_ and [DipR]_5_ can be used for the delivery of cargo GFP and RFP proteins.

To further explore the efficiency of [WR]_9_ in protein delivery, we also used fluorescent-labeled histone H2A. Histone proteins are involved in packaging DNA into nucleosomes. Histones maintain the shape and structure of a nucleosome [53,54]. Histone H2A is among the five main histone proteins responsible for the structure of chromatin in eukaryotic cells. Histones have been proposed as antimicrobial agents via the mediation of neutrophil extracellular traps (NETs), a first-line defense against many microorganisms [55,56,57,58,59]. Moreover, H2A disrupts DNA organization and stops the transcription of microorganisms upon cellular entry [60]. However, due to the size and hydrophilic/phobic nature of histones, they pose low cellular uptake.

**Figure 13:**
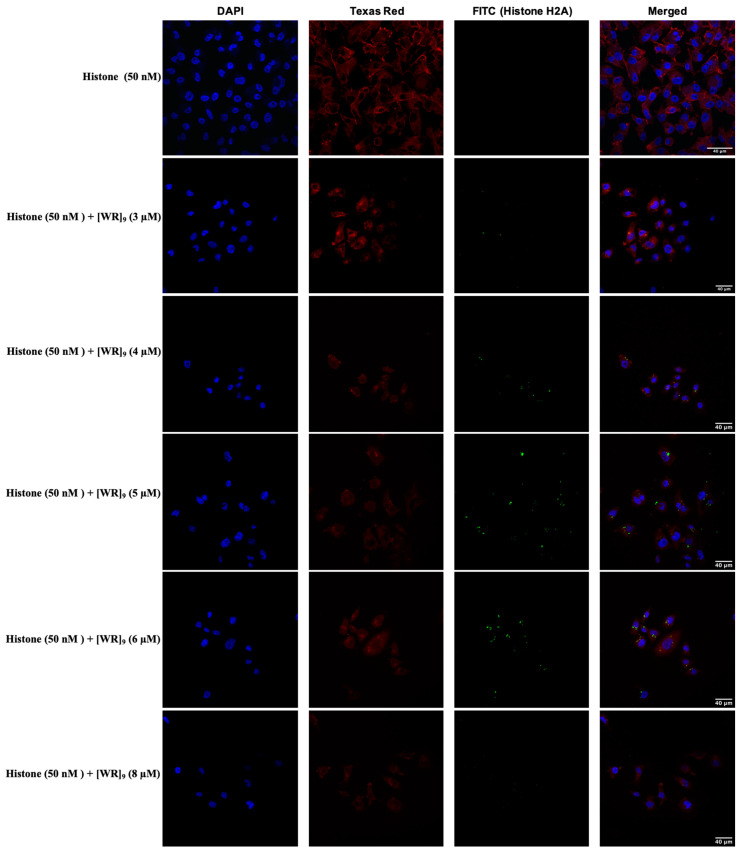
Confocal microscopy images of MDA-MB-231 cells incubated with the fluorescently labeled histone H2A-[WR]_9_ mixture at a peptide concentration (3–8 µM) and RFP at (50 nM) for 3 h. The blue, red, and green channels visualize DAPI (used to stain the nucleus), Texas Red (used to stain the cell membrane), and fluorescently labeled histone H2A, respectively.

Regardless of cellular composition and differences between cancer and bacterial cells, here we aimed to evaluate whether [WR]_9_ was able to deliver fluorescent-labeled histone H2A. Cells were incubated with the peptide at concentrations of 3–8 µM and fluorescent-labeled histone H2A at (50 nM). Confocal images revealed that most of the uptake occurred with peptide concentrations of 4, 5, and 6 µM, and the delivery was mainly in the cytoplasm (Figure 13).

These results open new venues for applying cyclic CPPs for the delivery of therapeutic proteins. At the same time, these avenues need to be explored further in protein uptake. Our laboratory is currently exploring other lead peptides for protein delivery applications.

**Figure 14:**
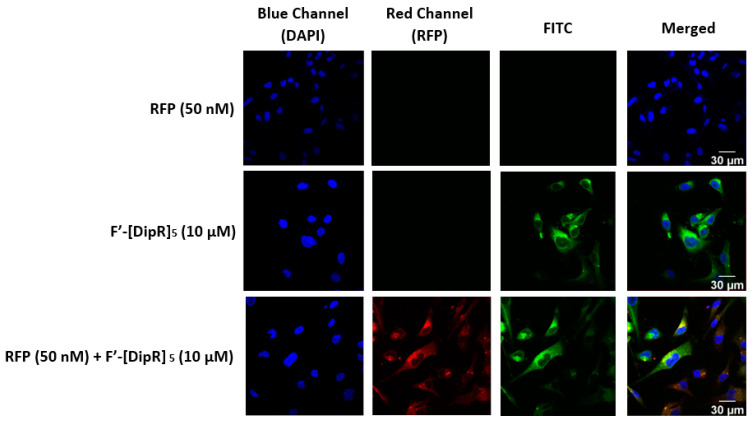
Confocal microscopy images of MDA-MB-231 cells incubated with the F′-[DipR]_5_ or RFP-F′-[DipR]_5_ mixture at a peptide concentration (10 µM) and RFP at (50 nM) for 3 h. The red and blue channels visualize RFP and DAPI (used to stain the nucleus), respectively.

**Figure 15:**
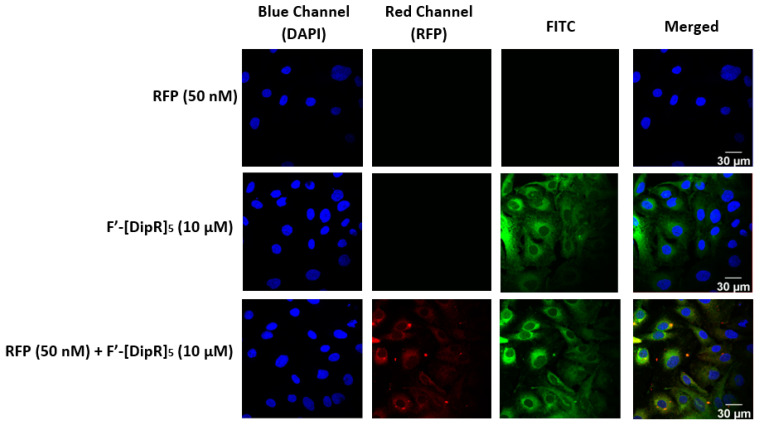
Confocal microscopy images of SK-OV-3 cells incubated with the F′-[DipR]_5_ or RFP-F′-[DipR]_5_ mixture at a peptide concentration (10 µM) and RFP at (50 nM) for 3 h. The red and blue channels visualize RFP and DAPI (used to stain the nucleus), respectively.

We have previously reported the synthesis and localization of fluorescence-labeled [KDipR]_5_ (F′-[DipR]_5_, Figure 1) in the cytosol [49]. We explored to determine whether the fluorescence signal for this peptide coalesces with the red fluorescence signal of RFP after incubation with MDA-MB-231 and SK-OV-3 cells. The data showed that both green and red fluorescence signals from fluorescence-labeled [KDipR]_5_ and RFP merge as yellow, suggesting colocalization of the peptide and protein and that the peptide indeed carries the protein to the cytosol (Figure 14 and Figure 15).

### 2.4. Fluorescent-Assisted Cell Sorting (FACS)

**Figure 16:**
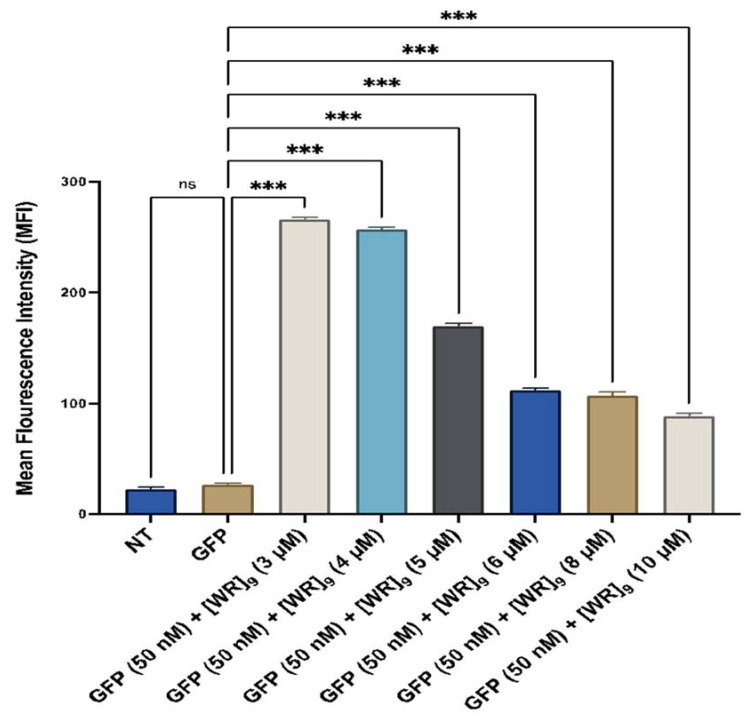
Cellular uptake of GFP-[WR]_9_ mixture at a peptide concentration (3–10 µM) and GFP at (50 nM) for 3 h in MDA-MB-231 studied by flow cytometry. The results are mean ± SD (*n* = 3) (ns; no significance, *** *p* < 0.001), treatment vs. Ctrl (GFP alone).

We used FACS to quantify GFP uptake using MDA-MB-231 cell line. Cells were incubated with [WR]_9_ at concentrations (3–10 µM) and GFP at (50 nM), as described above for 3 h. The results showed significant uptake (*p*-value < 0.001) with all peptide concentrations used. However, 3 and 4 µM of [WR]_9_ showed significantly higher GFP uptake compared to [WR]_9_ concentrations (5–10 µM). When compared to GFP alone, 3 and 4 µM of [WR]_9_ showed a 11- and 9.5-fold higher uptake, respectively, while [WR]_9_ (5–10 µM) showed a range of 6- to 3-fold increase in the uptake of GFP (Figure 16). These data were consistent with the confocal microscopy data shown in Figure 5 and Figure 6, with a high GFP uptake at concentrations of 3–4 µM. Although the uptake was also observed at higher concentrations, the cytotoxicity of [WR]_9_ was a limiting factor, as shown by cell morphological changes at 10 µM.

**Figure 17:**
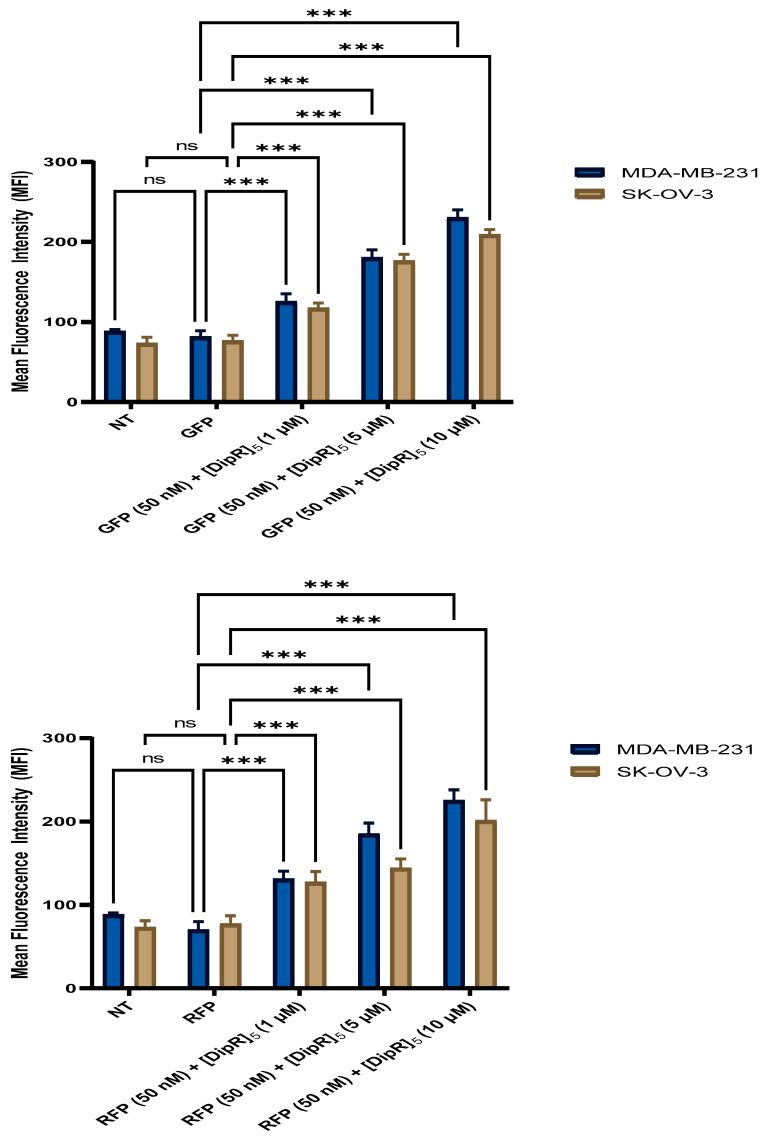
Cellular uptake of the GFP-[DipR]_5_ mixture at a peptide concentration (1–10 µM) and GFP or RFP at (50 nM) for 3 h in MDA-MB-231 and SK-OV-3 cells studied by flow cytometry. The results are mean ± SD (*n* = 3) (ns; no significance, *** *p* < 0.001), treatment vs. Ctrl (GFP alone).

Similar studies were conducted with [DipR]_5_ (1–10 µM) in combination with GFP (50 nM) or RFP (50 nM) in MDA-MB-231 and SK-OV-3 cells. The uptake in both cell lines was not significantly different. However, FACS studies demonstrated that [DipR]_5_ enhanced the uptake of both GFP and RFP, and the uptake was concentration-dependent, with the highest uptake at 10 µM (Figure 17). The data correlate well with confocal microscopy data for [DipR]_5_ with GFP (Figure 8 and Figure 9) and RFP (Figure 11 and Figure 12). As shown in confocal microscopy (Figure 15), F′-[DipR]_5_ enhanced the uptake of RFP. FACS studies were consistent with confocal microscopy (Figure S10, Supporting Information).

**Figure 18:**
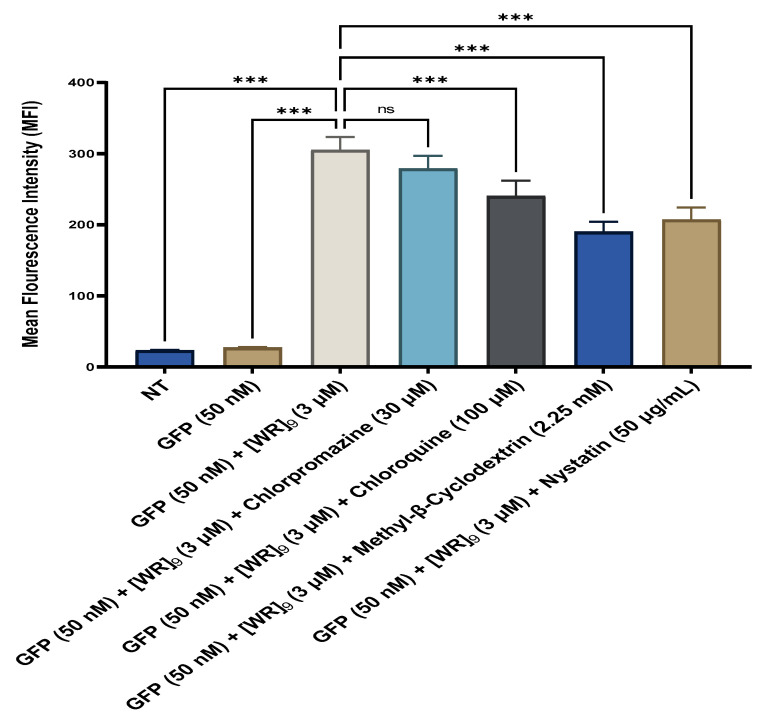
Cellular uptake of the GFP-[WR]_9_ mixture at a peptide concentration (3 µM) and GFP at (50 nM) for 3 h in MDA-MB-231 cells studied by flow cytometry. The results are mean ± SD (*n* = 3) (ns; no significance, *** p < 0.001); treatments vs. control (GFP alone).

**Figure 19:**
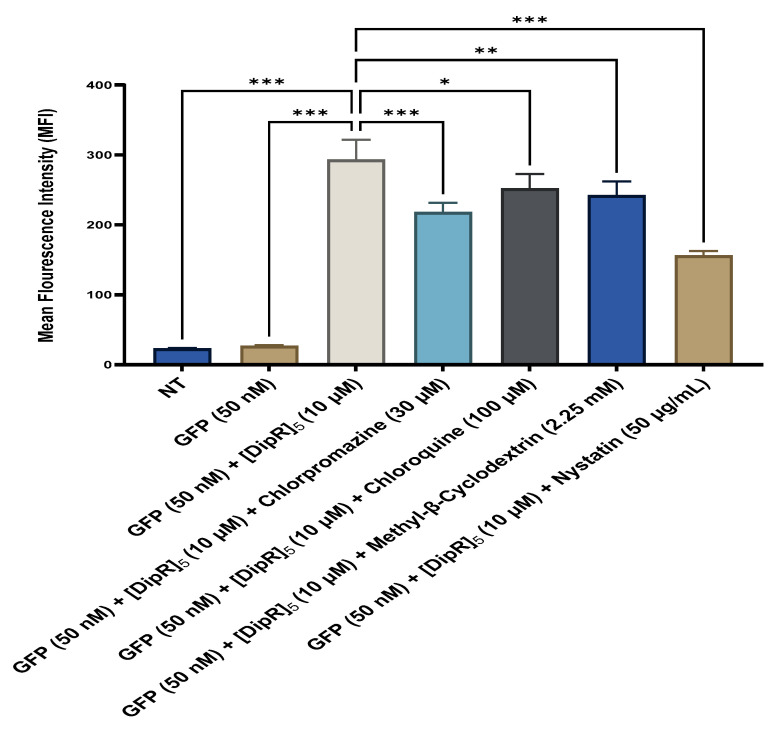
Cellular uptake of the GFP-[DipR]_5_ mixture at a peptide concentration (10 µM) and GFP at (50 nM) for 3 h in MDA-MB-231 cells studied by flow cytometry. The results are mean ± SD (*n* = 3) (ns; no significance, * *p* < 0.05, ** *p* < 0.01, *** *p* < 0.001; treatments vs. control (GFP alone).

Mechanistic studies were performed to determine whether the cellular uptake of the GFP in the presence of [WR]_9_ or [DipR]_5_ is endocytosis-dependent. These studies were conducted by confocal microscopy (Figure S11) and quantitated by flow cytometry in MDA-MB-231 cells to measure the uptake of GFP in the presence of peptides [WR]_9_ and [DipR]_5_ and various endocytosis inhibitors, including chlorpromazine, chloroquine, methyl-β-cyclodextrin, and nystatin. MDA-MB-231 cells were preincubated by various endocytosis inhibitors for 30 min. Then, the cells were incubated with GFP (50 nM) with [WR]_9_ (3 μM) or GFP (50 nM) with [DipR]_5_ (10 μM) for 3 h in the presence of endocytosis inhibitors (Figure 18 and Figure 19).

The cellular uptake of GFP (50 nM) in the presence of [WR]_9_ (3 μM) was not reduced significantly by chlorpromazine, which inhibits clathrin-mediated endocytosis. Chloroquine (30 μM) and nystatin (50 μg/mL) decreased the uptake of GFP (50 nM) and [WR]_9_ (3 μM) by 21 and 32%, respectively. None of the endocytosis inhibitors could completely stop the cellular uptake; however, a significant reduction in uptake was in the presence of methyl-β-cyclodextrin, which disrupts the lipid rafts of the cell membrane. The uptake of GFP (50 nM) in the presence of [WR]_9_ (3 μM) was slightly inhibited by methyl-β-cyclodextrin and nystatin endocytosis inhibitors after 3 h of incubation in MDA-MB-231 cells (Figure 18), suggesting the partial uptake through the caveolae/lipid-mediated endocytosis pathway. As a result, the combination of direct penetration and caveolae/lipid-mediated endocytosis may be involved in the uptake of GFP (50 nM) in the presence of [WR]_9_ (3 μM) across the cell membrane.

The cellular uptake of GFP (50 nM) in the presence of [DipR]_5_ (10 μM) was significantly reduced by all inhibitors. However, chloroquine, a clathrin-mediated endocytosis inhibitor, reduced it the least. Methyl-β-cyclodextrin (2.25 nM) and chlorpromazine (30 μM) decreased the uptake of GFP (50 nM) in the presence of [DipR]_5_ (10 μM) by 17 and 26%, respectively. Thus, the uptake of GFP in the presence of [DipR]_5_ was different from that of [WR]_9_. Even though none of the endocytosis inhibitors could completely stop the cellular uptake, a major reduction in uptake was observed in the presence of nystatin, which disrupts the caveolae/lipid-mediated endocytosis. The uptake of GFP (50 nM) and [DipR]_5_ (10 μM) was partially inhibited by nystatin and chlorpromazine after 3 h of incubation in MDA-MB-231 cells (Figure 19), suggesting a combination of multifaceted mechanisms. As a result, a combination of direct penetration and various endocytic processes presumably contribute to the uptake of GFP (50 nM) in the presence of [DipR]_5_ (10 μM) across the cell membrane.

## 3. Materials and Methods

### 3.1. Materials

All protected amino acids and resins were purchased from AAPPTEC (Louisville, KY, USA). All the other chemical reagents were purchased from Millipore Sigma (Milwaukee, WI, USA). Recombinant GFP and RFP were obtained from Novus Biologicals (Centennial, CO, USA). Histone 2A was provided by Dr. Albert Siryaporn from the University of California Irvine. Cell growth medium, fetal bovine serum, and all other cell biology reagents were purchased from Wilken Scientific (Pawtucket, RI, USA) and Fisher Scientific (Hanover Park, IL, USA). The final products were characterized by high-resolution matrix-assisted laser desorption/ionization time-of-flight (MALDI-TOF, GT 0264) from Bruker Inc. (Billerica, MA, USA) with α-cyano-4-hydroxycinnamic acid as a matrix. The final crude product was purified by a reversed-phase HPLC from Shimadzu (LC-20AP) (Canby, OR, USA) by using a gradient system of water and acetonitrile and a reversed-phase preparative column (XBridge BEH130 Prep C18 from Waters (Milford, MA, USA).

Human breast adenocarcinoma cells (MDA-MB-231, ATCC No. CRM-HTB-26) and human epithelial ovary adenocarcinoma cells (SK-OV-3, ATCC No. HTB-77) were obtained from American Type Culture Collection (ATCC, Manassas, VA, USA). VECTASHIELD VIBRANCE with DAPI (used to stain the cell nuclei) was obtained from Vector Laboratories (Burlingame, CA, USA). Cell Titer 96^®^ AQueous MTS Reagent was obtained from Promega (Madison, WI, USA). The MTS reagent was composed of a tetrazolium derivative (3-(4,5-dimethylthiazol-2-yl)-5-(3-carboxymethoxyphenyl)-2-(4-sulfophenyl)-2H-tetrazolium (named MTS) and phenazine ethosulfate (PES) and was used for the cell-based proliferation studies. All the materials for cell culture studies were purchased from Fisher Scientific (Hanover Park, IL, USA).

### 3.2. Synthesis of Peptides

The peptides were synthesized according to our previously reported procedure [38,39,45,46,47,48,49], using Fmoc/tBu solid-phase peptide synthesis and solution-phase cyclization. The molecular weight of all the peptides was confirmed with MALDI-TOF. The peptides were purified using reversed-phase HPLC and lyophilized. As a representative example, the molecular weight of [WR]_9_ is reported here. We have already reported other peptides [38,39,45,46,47,48,49].

[WR]_9_: MALDI-TOF (*m*/*z*): C_153_H_198_N_54_O_18,_ calculated: 3079.6238, found: 3080.6093 [M+H]^+^.

### 3.3. Cell Culture and Cytotoxicity Assay of [WR]_9_-Protein Physical Mixture and [DipR]_5_-Protein Physical Mixture

The cytotoxic activity of [WR]_9_ + GFP/RFP physical mixtures was evaluated in MDA-MB-231 cells, according to the previously reported procedure [38]. In brief, the cells were seeded at 5000 cells (0.1 mL per well in 96-well plates). An appropriate growth medium was used for each cell line (for MDA-MB-231 DMEM/F12 (1:1) (1x) with L-Glutamine and 15 mM HEPES containing FBS (10%) and penicillin or streptomycin (1%)). The cells were seeded in a complete growth medium 24 h prior to the experiment. The protein concentration was fixed at (50 nM). Different peptide concentrations ranging from 1 to 20 µM were added to each well in triplicate. Then, the cells were incubated for 24 h at 37 °C in a humidified atmosphere of 5% CO_2_. After the incubation period, MTS reagent (20 µL) was added to each well. The incubation was continued for 3 h. The MTS protocol is based on the reduction reaction of MTS tetrazolium by the viable cells. Cell viability was then measured by the determination of the fluorescence intensity at 490 nm using a SpectraMax M2 microplate spectrophotometer. The percentage of cell viability was then calculated using the following equation: [(OD value of cells treated with the compound) − (OD value of culture medium)]/[(OD value of control cells) − (OD value of culture medium)] × 100%.

### 3.4. Confocal Microscopy

MDA-MB-231 and SK-OV-3 cells (7 × 10^4^ cells/well) were seeded with a medium on a coverslip 24 h prior to the experiment in 6-well plates. After 24 h, the medium was changed, and cells were treated with protein–peptide physical mixtures using the method explained for flow cytometry experiments (below). Peptide/protein physical mixtures were prepared using serum-free media as a diluent to achieve a final concentration of 50 nM for GFP, RFP, and histone H2A proteins and different peptide concentrations. Cells exposed to the mixtures were incubated at 37 ºC and standard growth conditions for 3 h. After 3 h, the growth media was removed, and cells were washed with clear HBSS. The cells were then fixed using 3.7% formaldehyde solution in HBSS for about 10 min and were then exposed to Texas Red for 60 min and DAPI overnight, away from light, at room temperature. Texas red and DAPI are used to stain cell membranes and nuclei, respectively. Coverslips were examined using a Nikon A1R high-definition resonant scanning confocal microscope and NIS-Elements software (AR 4.30.02, 64 bit).

### 3.5. Cellular Internalization and Mechanistic Studies (Flow Cytometry)

The efficiency of the peptides in internalizing model protein (GFP) into human cells was evaluated by quantifying the uptake of fluorescence by flow cytometry (BD-FACSVerse; BD Biosciences; San Jose, CA, USA). MDA-MB-231 and SK-OV-3 cells were used for this study and were seeded in 24-well plates (~200,000 cells per well). The peptide/protein mixtures were prepared with a final concentration of 50 nM for GFP or RFP protein and different peptide concentrations. Cells exposed to the mixtures were incubated at 37 °C and standard growth conditions for 3 h. Then, the cells were washed with clear HBSS, trypsinized, and fixed using 3.7% formaldehyde solution. Suspended cells were analyzed using the FITC channel to quantify cell-associated fluorescence for GFP or RFP. The uptake analysis was processed according to the calibration of the signal gated from the non-treated cells (as the negative control). The mixture was incubated for 30 min before any assays to obtain a satisfactory binding.

Mechanistic studies were performed in MDA-MB-231 cells to determine the mechanism of the cellular uptake of GFP in the presence of [WR]_9_ or [DipR]_5_. The cells were incubated with various endocytosis inhibitors, including chlorpromazine (30 μM), chloroquine (100 μM), methyl-β-cyclodextrin (2.25 mM), and nystatin (50 μg/mL) for 30 min. Simultaneously, the GFP and peptide physical mixtures were left for 30 min. The cells were then incubated with GFP and [WR]_9_ or [DipR]_5_ for 3 h. The flow cytometry study was performed as mentioned above.

### 3.6. Data Analysis

The data are presented as the mean standard deviation for the stated number of samples. A significant difference test was performed using a Student’s *t*-test between the two groups. For data over 3 groups, one-way ANOVA followed by post hoc Tukey tests were performed. The alpha threshold was set to 0.05 with a 95% confidence interval.

## 4. Conclusions

In a continuous effort to explore cyclic CPPs applications, our laboratory investigated the ability of a number of cyclic and hybrid cyclic–linear peptides, [WR]_4_, [WR]_9_, [WWRR]_5_, [WWRR]_4_, [(RW)_5_K](RW)_5_, [R_5_K]W_7_, and [DipR]_5_, for the delivery of proteins (e.g., GFP or RFP) in MDA-MB-231 and SK-OV-3 cells. Confocal microscopy showed that [WR]_9_ and [DipR]_5_ were the most efficient among all the peptides for the delivery of proteins. Thus, these two peptides were selected for further cell-based studies. The physical mixture of [WR]_9_ and [DipR]_5_ with the selected protein (GFP or RFP) physical mixture did not show high cytotoxicity after 24 h of incubation. [WR]_9_ and [DipR]_5_ were capable of internalization of two protein models, GFP and RFP. [WR]_9_ was also shown to improve the delivery of histone H2A. The mechanistic studies revealed that a combination of mechanisms contributes to the uptake of GFP (50 nM) in the presence of [WR]_9_ (3 μM) or [DipR]_5_ (10 μM) across the cell membrane. These results open new avenues for the application of CPPs for the delivery of therapeutic proteins. While these avenues need to be explored further to determine the function of delivered proteins, our laboratory is currently exploring other lead peptides for protein delivery applications. We have already conducted CPP-drug conjugates that showed the effectiveness of the covalent strategy for small drug molecules, such as doxorubicin [47,52,61,62], camptothecin, and paclitaxel [63]. Thus, future steps include applying chemical conjugation of lead peptides with proteins of therapeutic relevance. Further studies are needed to assess their potential as a non-toxic protein delivery agent.

## Data Availability

The data presented in this study are available on request from the corresponding author.

## Supplementary Materials

The following supporting information can be downloaded at: https://www.mdpi.com/article/10.3390/ph16030469/s1, Table S1. Cytotoxicity of [WR]_9_ with GFP (50 nM) physical mixtures; Table S2. Cytotoxicity of [WR]_9_ with RFP (50 nM) at different peptide concentrations; Table S3. Cytotoxicity of [DipR]_5_, [WWRR]_4_, and [WWRR]_5_ with GFP (50 nM) at different peptide concentrations (1–10 µM) in breast cancer (MDA-MB-231) cell line after 3 h as determined by MTS assay; Table S4. Cytotoxicity of [WR]_9_ (3 μM) with increasing GFP concentrations (100–500 nM) in breast cancer (MDA-MB-231) cell line after 24 h as determined by MTS assay; Table S5. Cytotoxicity of [DipR]_5_ (10 μM) with increasing GFP concentrations (100–500 nM) in breast cancer (MDA-MB-231) cell line after 3 h as determined by MTS assay; Figure S1. Cytotoxicity of cyclic peptides [WWRR]_4_ and [WWRR]_5_ with GFP (50 nM) mixtures at different peptide concentrations (1–10 µM) in breast cancer (MDA-MB-231) cell line after 3 h; Figure S2. Cytotoxicity of [WR]_9_ (3 μM) with increasing GFP concentrations (100–500 nM) in breast cancer (MDA-MB-231) cell line after 24 h as determined by MTS assay; Figure S3. Cytotoxicity of [DipR]_5_ (10 μM) with increasing GFP concentrations (100–500 nM) in breast cancer (MDA-MB-231) cell line after 3 h as determined by MTS assay; Figure S4. Confocal microscopy images of MDA-MB-231 cells incubated with GFP-[WR]_4_ mixture at a peptide concentration ranging (1–10 µM) and GFP at (50 nM) for 3 h; Figure S5. Confocal microscopy images of SK-OV-3 cells incubated with GFP-[WR]_4_ mixture at a peptide concentration range (1–5 µM) and GFP at (50 nM) for 3 h; Figure S6. Confocal microscopy images of MDA-MB-231 cells incubated with GFP-[R_5_K]W_7_ mixture at a peptide concentration range (1–5 µM) and GFP at (50 nM) for 3 h; Figure S7. Confocal microscopy images of MDA-MB-231 cells incubated with GFP-[(RW)_5_K](RW)_5_ mixture at a peptide concentration range (1–5 µM) and GFP at (50 nM) for 3 h; Figure S8. Confocal microscopy images of MDA-MB-231 cells incubated with GFP-[WWRR]_4_ mixture at a peptide concentration range (1–6 µM) and GFP at (50 nM) for 3 h; Figure S9. Confocal microscopy images of MDA-MB-231 cells incubated with GFP-[WWRR]_5_ mixture at a peptide concentration range (1–5 µM) and GFP at (50 nM) for 3 h; Figure S10. Improved delivery of RFP (50 nM) by F′-[DipR]5 in MDA-MB-231 cells; Figure S11. Cellular uptake study of GFP (50 nM) with [WR]_9_ in the presence of endocytosis inhibitors in MDA-MB-231 cells after 3 h incubation; Figure S12. Cellular uptake study of GFP (50 nM) with [DipR]_5_ in the presence of endocytosis inhibitors in MDA-MB-231 cells after 3 h incubation.
