# Supplementary material for: Amphiphilic Cell-Penetrating Peptides Containing Arginine and Hydrophobic Residues as Protein Delivery Agents
Source: Pharmaceuticals (Basel). 2023 Mar 22;16(3):469. doi: 10.3390/ph16030469 (PMC10053436; doi:10.3390/ph16030469)
Supplement: Supplementary file 1 [file pharmaceuticals-16-00469-s001.zip › pharmaceuticals-2288168-supplementary.pdf]

Supporting Information

Amphiphilic Cyclic Cell-Penetrating Peptides Containing Tryptophan and Arginine as Protein Delivery Agents

Jonathan Moreno[1,†], Khalid Zoghebi[1,2,†], David Salehi[1], Lois Kim[1], Sorour Khayyatnejad Shoushtari[1], Rakesh K Tiwari[1], Keykavous Parang[1]*

[1]Center for Targeted Drug Delivery, Department of Biomedical and Pharmaceutical Sciences, Chapman University School of Pharmacy, Harry and Diane Rinker Health Science Campus, Irvine, CA 92618, United States; jonmoreno@chapman.edu (J.M.); zoghebi@chapman.edu (K.Z.); dsalehi@chapman.edu (D.S.); lokim@chapman.edu (L. K.); khayyatnejadshoushta@chapman.edu (S.K.S.); tiwari@chapman.edu (R.K.T.); parang@chapman.edu (K.P.)

[2]Department of Pharmaceutical Chemistry, College of Pharmacy, Jazan University, Jazan 82826, Saudi Arabia; Kzoghebi@jazanu.edu.sa

Corresponding Author

Keykavous Parang − Center for Targeted Drug Delivery, Department of Biomedical and Pharmaceutical Sciences, Chapman University School of Pharmacy, Irvine, California92618, United States; orcid.org/0000-0001-8600-0893; Phone: +1-714-516-5489; Email: parang@chapman.edu; Fax: +1-714-516-5481.

†These authors contributed equally.

Table of Contents	Page
**Table S1.** Cytotoxicity of $[WR]_9$ with GFP (50 nM) physical mixtures.	S4
**Table S2.** Cytotoxicity of $[WR]_9$ with RFP (50 nM) at different peptide concentrations.	S5
**Table S3.** Cytotoxicity of $[DipR]_5$, $[WWRR]_4$, and $[WWRR]_5$ with GFP (50 nM) at different peptide concentrations (1-10 µM) in breast cancer (MDA-MB-231) cell line after 3 h as determined by MTS assay.	S6
**Figure S1.** Cytotoxicity of cyclic peptides $[WWRR]_4$ and $[WWRR]_5$ with GFP (50 nM) complexes at different peptide concentrations (1-10 µM) in breast cancer (MDA-MB-231) cell line after 3 h.	S6
**Table S4.** Cytotoxicity of $[WR]_9$ (3 µM) with increasing GFP concentrations (100 nM – 500 nM) in breast cancer (MDA-MB-231) cell line after 24 h as determined by MTS assay.	S7
**Figure S2.** Cytotoxicity of $[WR]_9$ (3 µM) with increasing GFP concentrations (100 nM – 500 nM) in breast cancer (MDA-MB-231) cell line after 24 h as determined by MTS assay.	S7
**Table S5.** Cytotoxicity of $[DipR]_5$ (10 µM) with increasing GFP concentrations (100 nM – 500 nM) in breast cancer (MDA-MB-231) cell line after 3 h as determined by MTS assay.	S8
**Figure S3.** Cytotoxicity of $[DipR]_5$ (10 µM) with increasing GFP concentrations (100 nM – 500 nM) in breast cancer (MDA-MB-231) cell line after 3 h as determined by MTS assay.	S8
**Figure S4.** Confocal microscopy images of MDA-MB-231 cells incubated with GFP-$[WR]_4$ mixture at a peptide concentration ranging (1-10 µM) and GFP at (50 nM) for 3 h.	S9
**Figure S5.** Confocal microscopy images of SK-OV-3 cells incubated with GFP-$[WR]_4$ mixture at a peptide concentration range (1-5 µM) and GFP at (50 nM) for 3 h.	S10
**Figure S6.** Confocal microscopy images of MDA-MB-231 cells incubated with GFP-$[R_5K]W_7$ mixture at a peptide concentration range (1-5 µM) and GFP at (50 nM) for 3 h.	S11
**Figure S7.** Confocal microscopy images of MDA-MB-231 cells incubated with GFP-$[(RW)_5K](RW)_5$ mixture at a peptide concentration range (1-5 µM) and GFP at (50 nM) for 3 h.	S12

**Figure S8.** Confocal microscopy images of MDA-MB-231 cells incubated with GFP-$[WWRR]_4$ mixture at a peptide concentration range (1-6 µM) and GFP at (50 nM) for 3 h.	S13
**Figure S9.** Confocal microscopy images of MDA-MB-231 cells incubated with GFP-$[WWRR]_5$ mixture at a peptide concentration range (1-5 µM) and GFP at (50 nM) for 3 h.	S14
**Figure S10.** Improved delivery of RFP (50 nM) by F′-[DipR]5 in MDA-MB-231 cells.	S15
**Figure S11.** Cellular uptake study of GFP (50 nM) with $[WR]_9$ in the presence of endocytosis inhibitors in MDA-MB-231 cells after 3 h incubation.	S16
**Figure S12.** Cellular uptake study of GFP (50 nM) with $[DipR]_5$ in the presence of endocytosis inhibitors in MDA-MB-231 cells after 3 h incubation.	S17

Table S1. Cytotoxicity of $[WR]_9$ with GFP (50 nM) at different peptide concentrations (1-20 µM) in breast cancer (MDA-MB-231) cell line after 24 h as determined by MTS assay.

**Compound**	**Cell Viability (%) after 24 h**
Non-treated Cells	100 ± 2
GFP (50 nM)	101 ± 3
GFP (50 nM) + $[WR]_9$ (1 µM)	99 ± 2
GFP (50 nM) + $[WR]_9$ (5 µM)	97 ± 5
GFP (50 nM) + $[WR]_9$ (10 µM)	94 ± 3
GFP (50 nM) + $[WR]_9$ (15 µM)	71 ± 2
GFP (50 nM) + $[WR]_9$ (20 µM)	60 ± 2

Table S2. Cytotoxicity of $[WR]_9$ with RFP (50 nM) at different peptide concentrations (1-20 μM) in breast cancer (MDA-MB-231) cell line after 24 h, as determined by MTS assay.

Compound	Cell Viability (%) after 24 h
Non-treated Cells	100 ± 1
RFP (50 nM)	98 ± 2
RFP (50 nM) + $[WR]_9$ (1 μM)	101 ± 5
RFP (50 nM) + $[WR]_9$ (5 μM)	96 ± 4
RFP (50 nM) + $[WR]_9$ (10 μM)	90 ± 2
RFP (50 nM) + $[WR]_9$ (15 μM)	73 ± 2
RFP (50 nM) + $[WR]_9$ (20 μM)	58 ± 2

Table S3. Cytotoxicity of $[DipR]_5$, $[WWRR]_4$, and $[WWRR]_5$ with GFP (50 nM) at different peptide concentrations (1-10 µM) in breast cancer (MDA-MB-231) cell line after 3 h as determined by MTS assay.

Compound	Cell Viability (%) after 3 h
Non-treated Cells	100 ± 1
GFP (50 nM)	101 ± 1
GFP (50 nM) + $[DipR]_5$ (1 µM)	83 ± 4
GFP (50 nM) + $[DipR]_5$ (10 µM)	81 ± 4
GFP (50 nM) + $[WWRR]_4$ (6 µM)	76 ± 3
GFP (50 nM) + $[WWRR]_5$ (5 µM)	76 ± 5

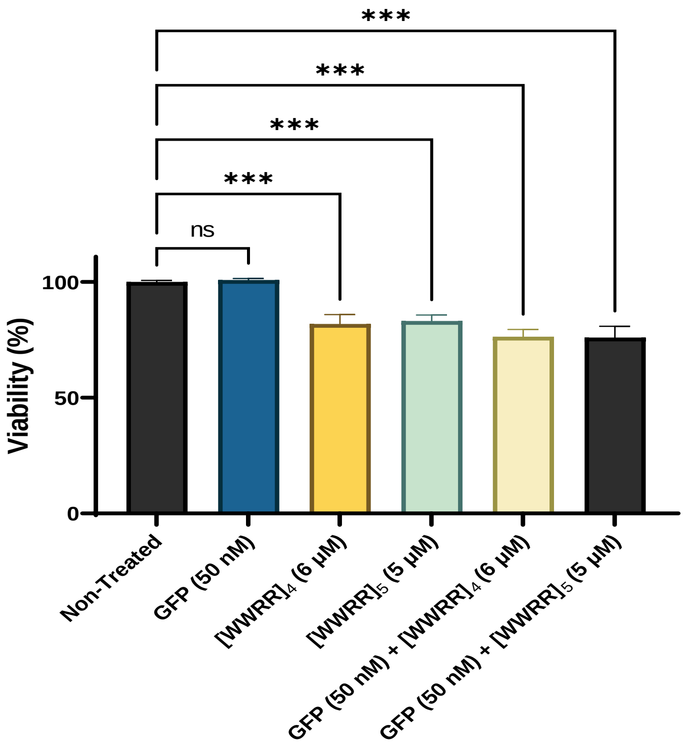

Figure S1. Cytotoxicity of cyclic peptides $[WWRR]_4$, and $[WWRR]_5$ with GFP (50 nM) complexes at different peptide concentrations (1-10 µM) in breast cancer (MDA-MB-231) cell line after 3 h. Results are mean ± SD (*n* = 3) (ns; no significance, *** $p < 0.001$ treatments vs. Ctrl (NT).

Table S4. Cytotoxicity of [WR]$_9$ (3 µM) with increasing GFP concentrations (100 nM – 500 nM) in breast cancer (MDA-MB-231) cell line after 24 h as determined by MTS assay.

Compound	Cell Viability (%) after 24 h
Non-treated Cells	100 ± 1
GFP (50 nM)	101 ± 1
GFP (100 nM)	103 ± 3
GFP (250 nM)	102 ± 2
GFP (500 nM)	98 ± 2
GFP (50 nM) + [WR]$_9$ (3 µM)	100 ± 3
GFP (100 nM) + [WR]$_9$ (3 µM)	100 ± 6
GFP (250 nM) + [WR]$_9$ (3 µM)	96 ± 5
GFP (500 nM) + [WR]$_9$ (3 µM)	95 ± 7

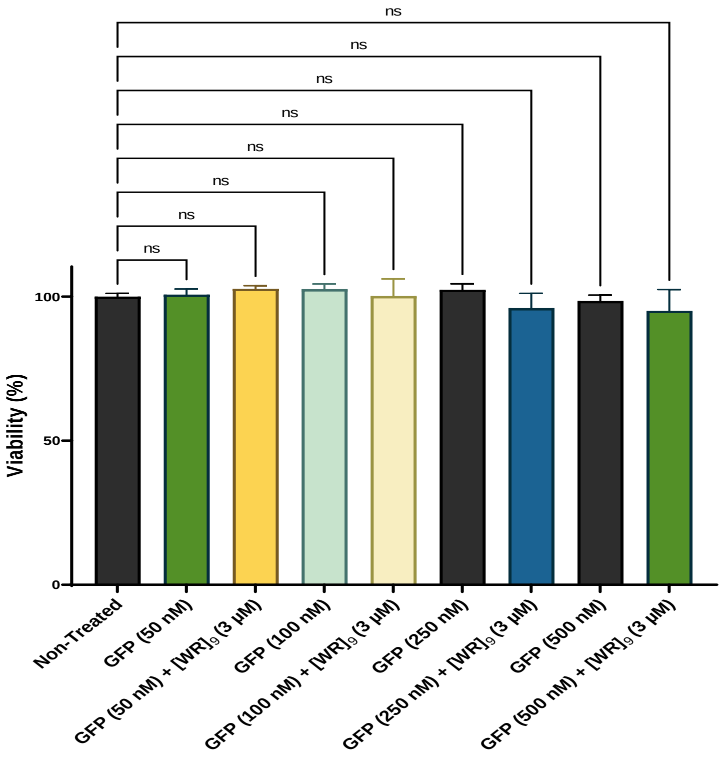

Figure S2. Cytotoxicity of [WR]$_9$ (3 µM) with increasing GFP concentrations (100 nM – 500 nM) in breast cancer (MDA-MB-231) cell line after 24 h as determined by MTS assay. Results are mean ± SD (n = 3) (ns; no significance) vs. Ctrl (NT).

Table S5. Cytotoxicity of $[DipR]_5$ (10 µM) with increasing GFP concentrations (100 nM – 500 nM) in breast cancer (MDA-MB-231) cell line after 3 h as determined by MTS assay.

Compound	Cell Viability (%) after 3 h
Non-treated Cells	100 ± 1
GFP (50 nM)	101 ± 1
GFP (100 nM)	103 ± 3
GFP (250 nM)	102 ± 2
GFP (500 nM)	98 ± 2
GFP (50 nM) + $[DipR]_5$ (10 µM)	81 ± 6
GFP (100 nM) + $[DipR]_5$ (10 µM)	88 ± 3
GFP (250 nM) + $[DipR]_5$ (10 µM)	91 ± 7
GFP (500 nM) + $[DipR]_5$ (10 µM)	84 ± 3

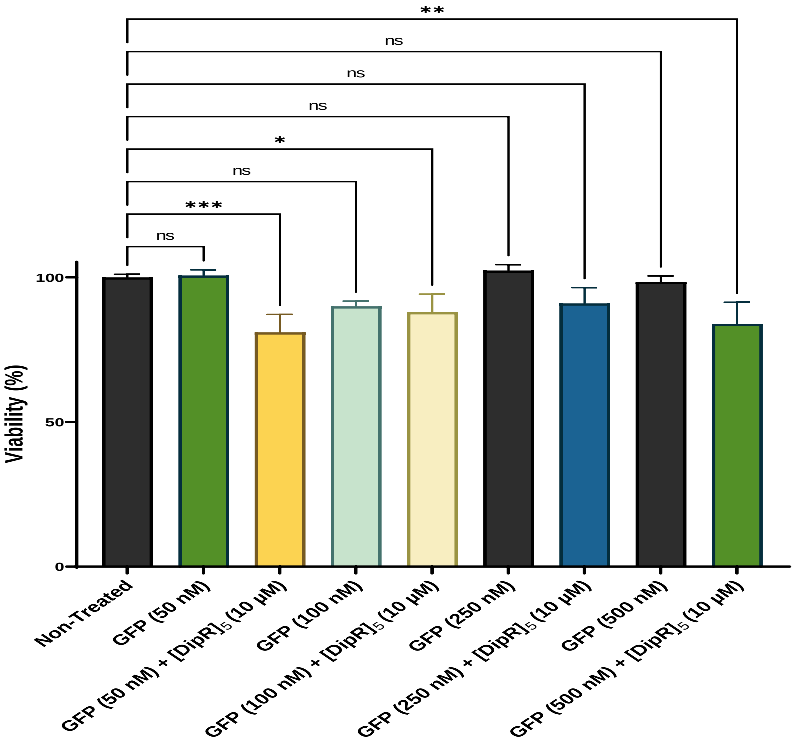

Figure S3. Cytotoxicity of $[DipR]_5$ (10 µM) with increasing GFP concentrations (100 nM – 500 nM) in breast cancer (MDA-MB-231) cell line after 3 h as determined by MTS assay. Results are mean ± SD (n = 3) (ns; no significance, * $p < 0.05$, ** $p < 0.01$, *** $p < 0.001$ treatments vs. Ctrl (NT).

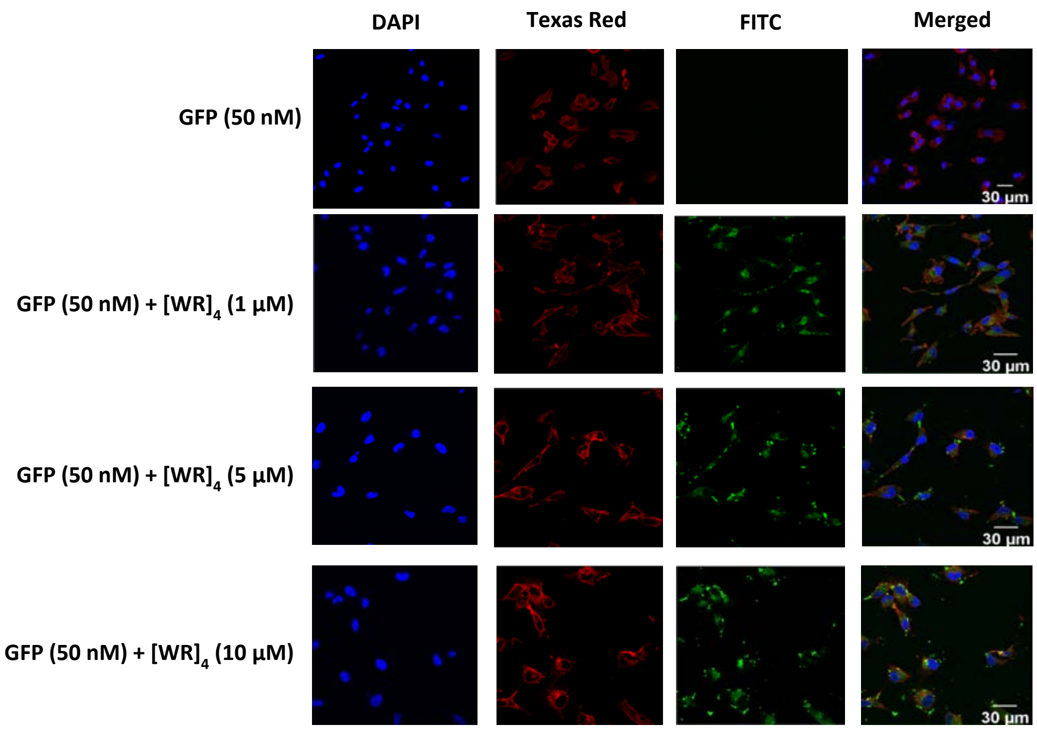

Figure S4. Confocal microscopy images of MDA-MB-231 cells incubated with GFP-$[WR]_4$ mixture at a peptide concentration ranging (1-10 µM) and GFP at (50 nM) for 3 h. The red, blue, and green channels visualize Texas Red (used to stain the cellular cytoskeleton), DAPI (used to stain the nucleus), and GFP, respectively.

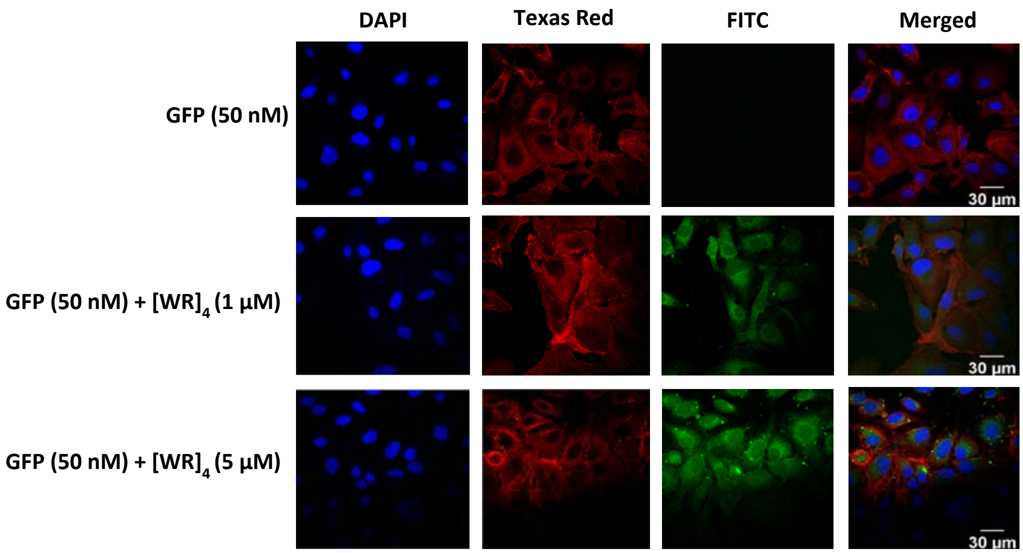

Figure S5. Confocal microscopy images of SK-OV-3 cells incubated with GFP-$[WR]_4$ mixture at a peptide concentration ranged (1-5 µM) and GFP at (50 nM) for 3 h. The red, blue, and green channels visualize Texas Red (used to stain the cellular cytoskeleton), DAPI (used to stain the nucleus), and GFP, respectively.

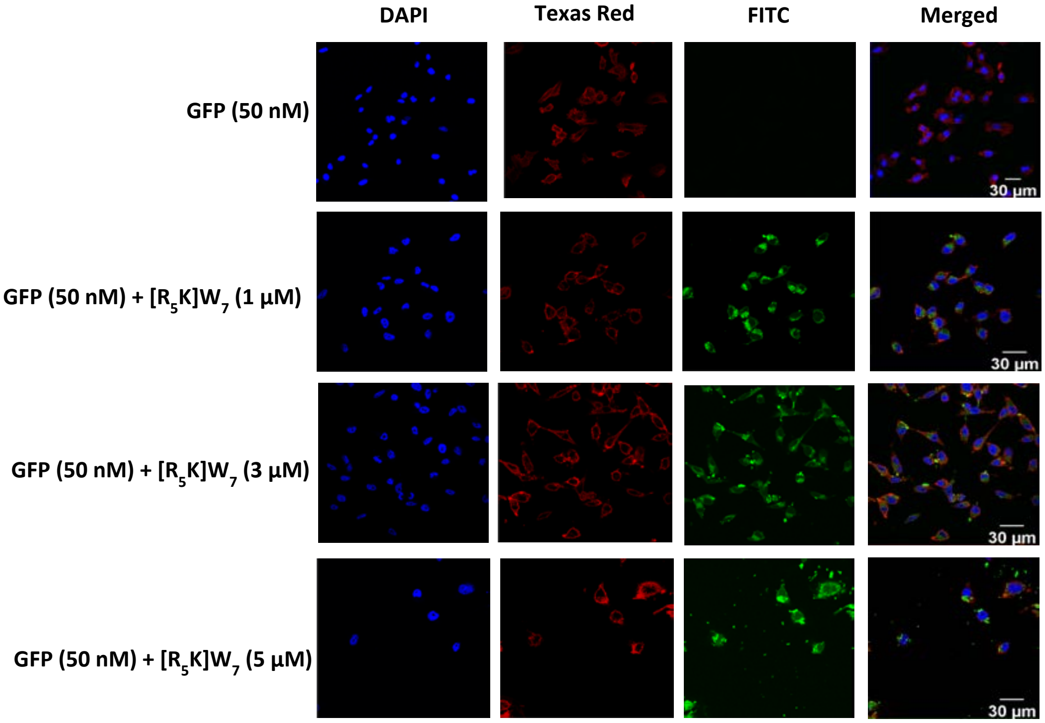

Figure S6. Confocal microscopy images of MDA-MB-231 cells incubated with GFP-$[R_5K]W_7$ mixture at a peptide concentration ranged (1-5 µM) and GFP at (50 nM) for 3 h. The red, blue, and green channels visualize Texas Red (used to stain the cellular cytoskeleton), DAPI (used to stain the nucleus), and GFP, respectively.

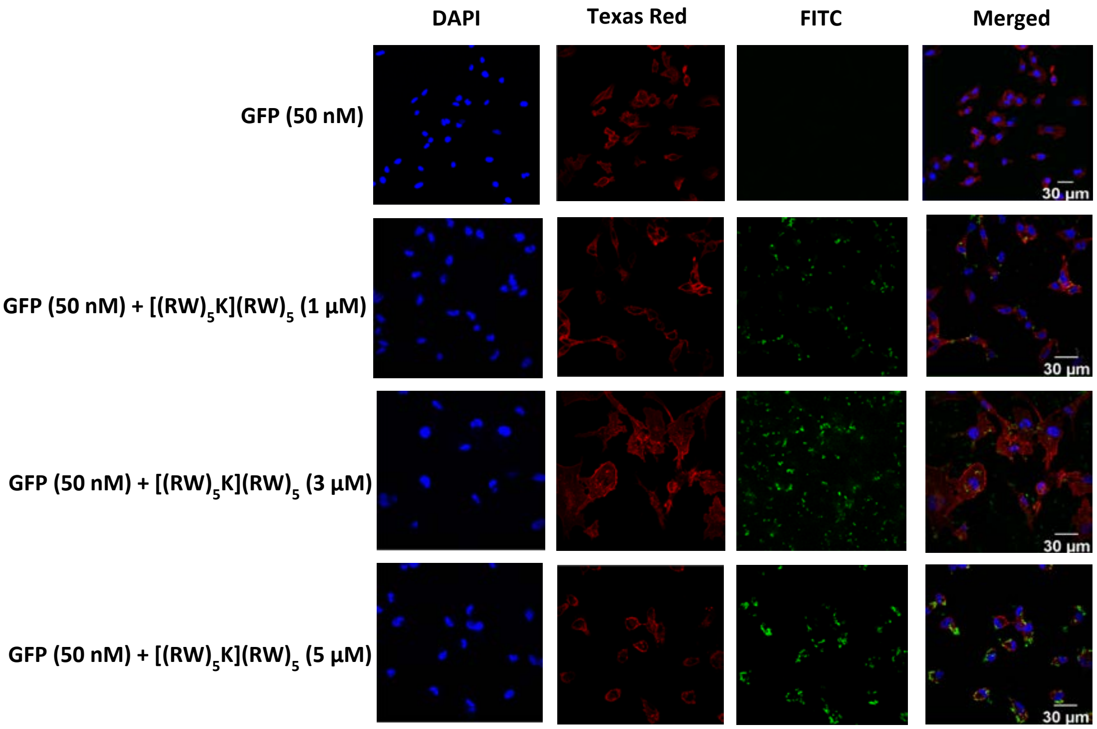

Figure S7. Confocal microscopy images of MDA-MB-231 cells incubated with GFP-$[(RW)_5K](RW)_5$ mixture at a peptide concentration ranged (1-5 µM) and GFP at (50 nM) for 3 h. The red, blue, and green channels visualize Texas Red (used to stain the cellular cytoskeleton), DAPI (used to stain the nucleus), and GFP, respectively.

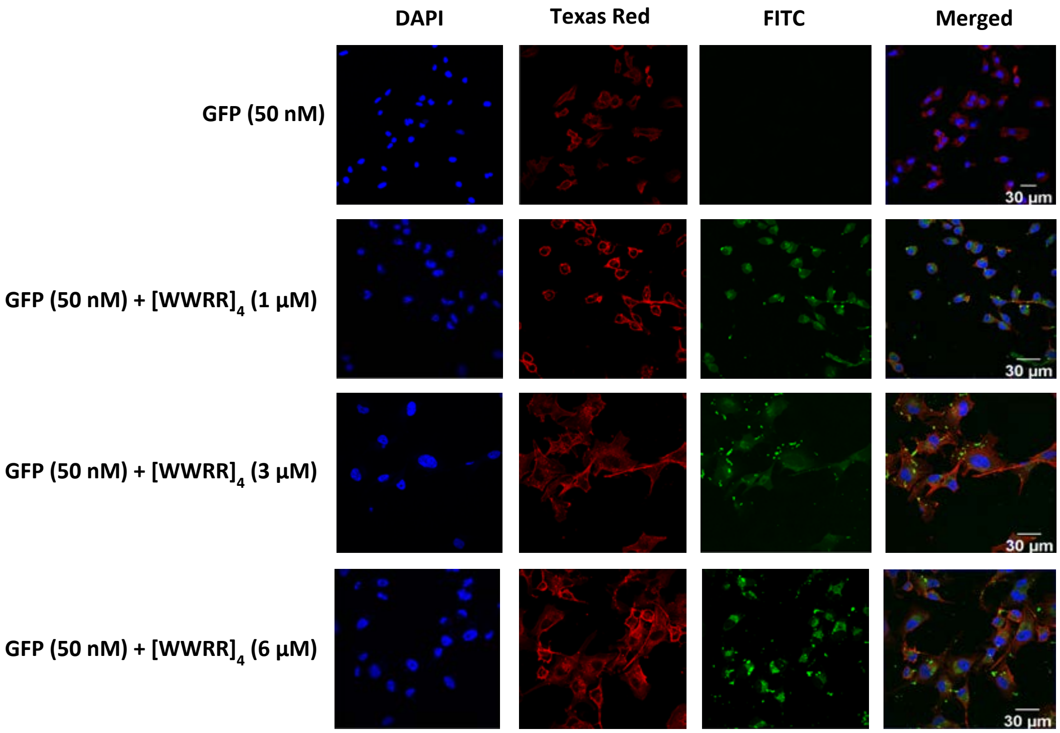

Figure S8. Confocal microscopy images of MDA-MB-231 cells incubated with GFP-[WWRR]$_4$ mixture at a peptide concentration ranging (1-6 µM) and GFP at (50 nM) for 3 h. The red, blue, and green channels visualize Texas Red (used to stain the cellular cytoskeleton), DAPI (used to stain the nucleus), and GFP, respectively.

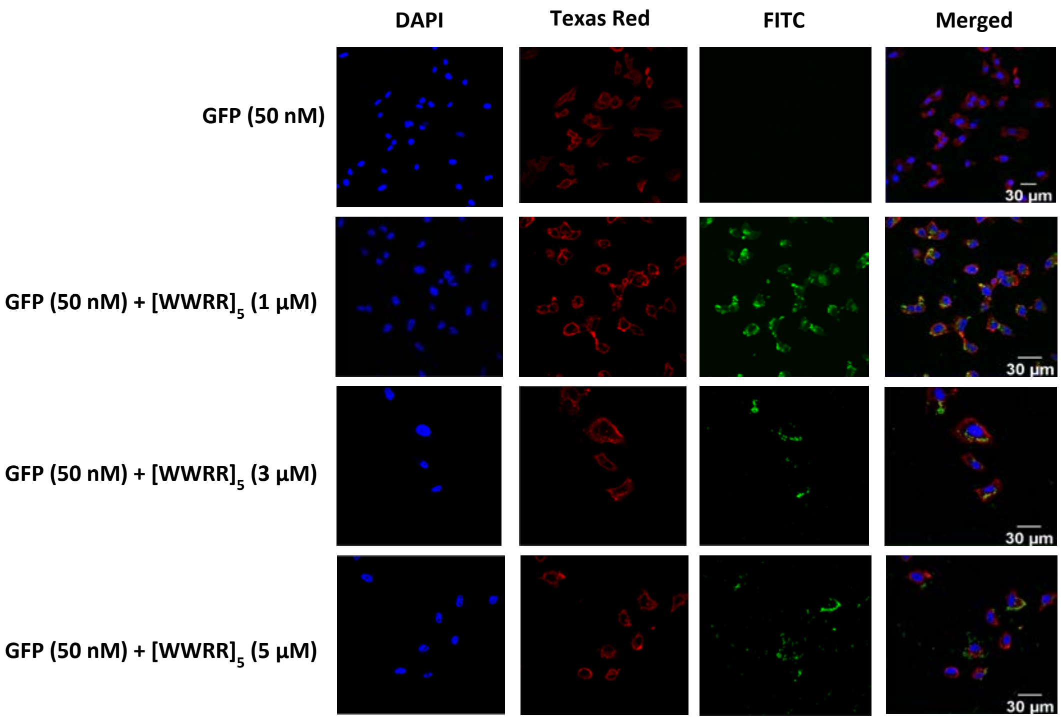

Figure S9. Confocal microscopy images of MDA-MB-231 cells incubated with GFP-[WWRR]$_5$ mixture at a peptide concentration ranging (1-5 µM) and GFP at (50 nM) for 3 h. The red, blue, and green channels visualize Texas Red (used to stain the cellular cytoskeleton), DAPI (used to stain the nucleus), and GFP, respectively.

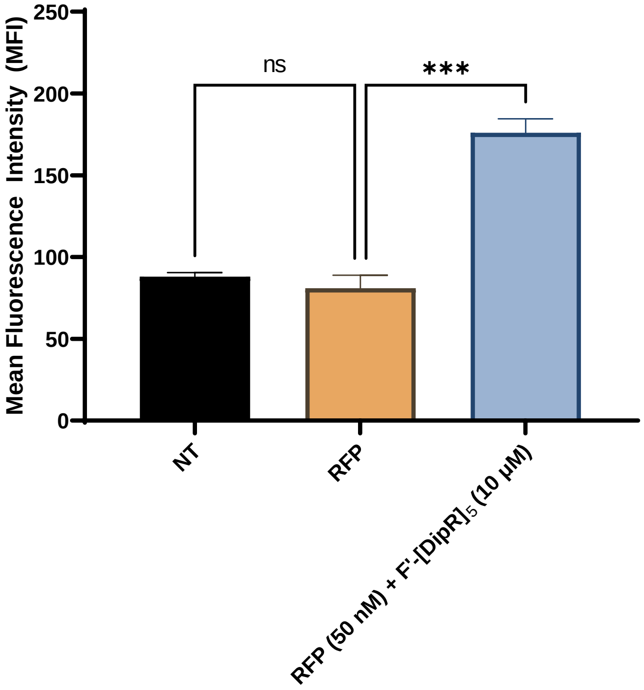

Figure S10. Improved delivery of RFP (50 nM) by F′-$[DipR]_5$ in MDA-MB-231 cells. Results are mean ± SD (n = 3) (ns; no significance, ****p*<0.001), treatment vs. Ctrl (RFP alone).

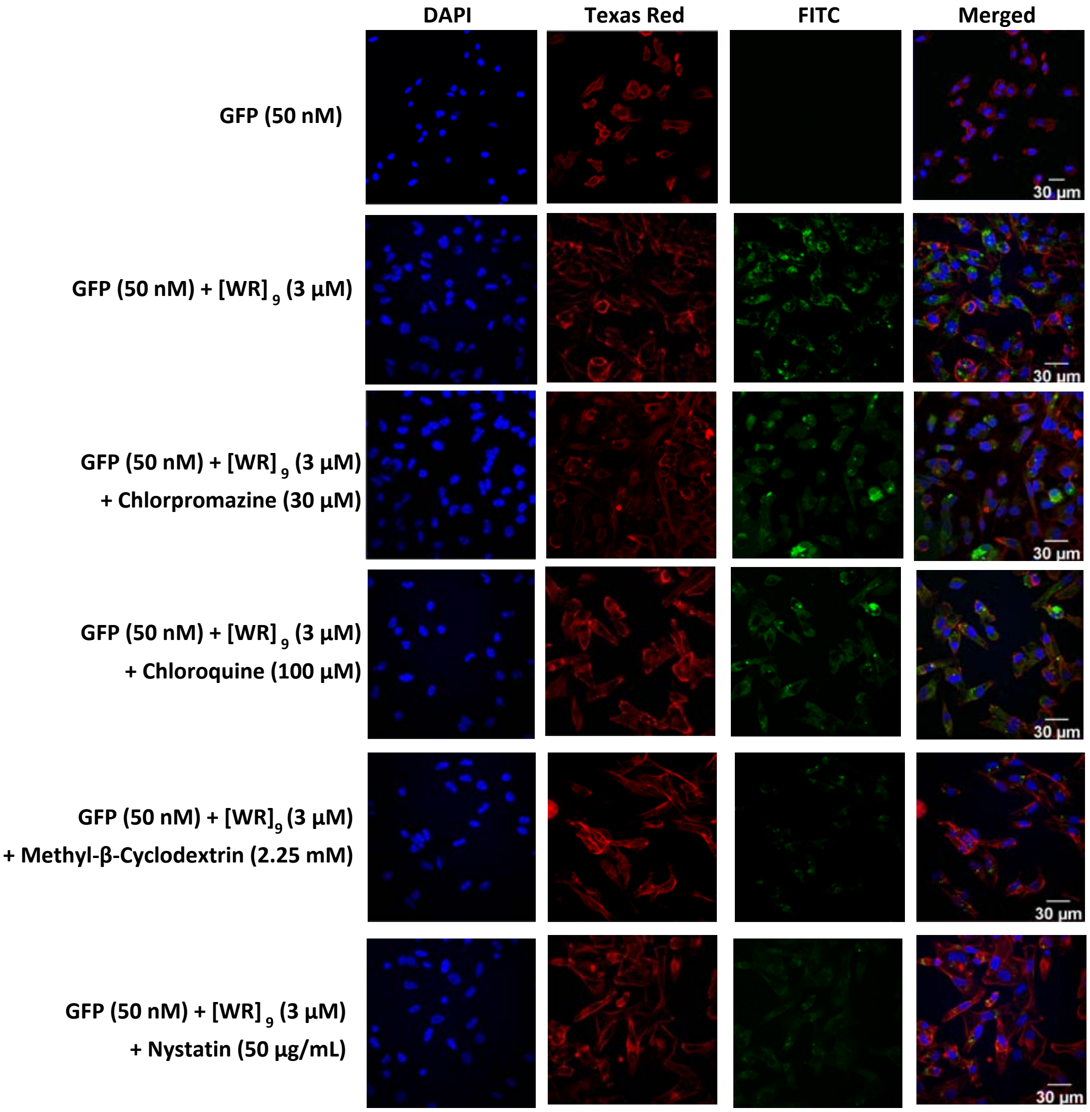

Figure S11. Cellular uptake study of GFP (50 nM) with $[WR]_9$ in the presence of endocytosis inhibitors in MDA-MB-231 cells after 3 h incubation. The red, blue, and green channels visualize Texas Red (used to stain the cellular cytoskeleton), DAPI (used to stain the nucleus), and GFP, respectively.

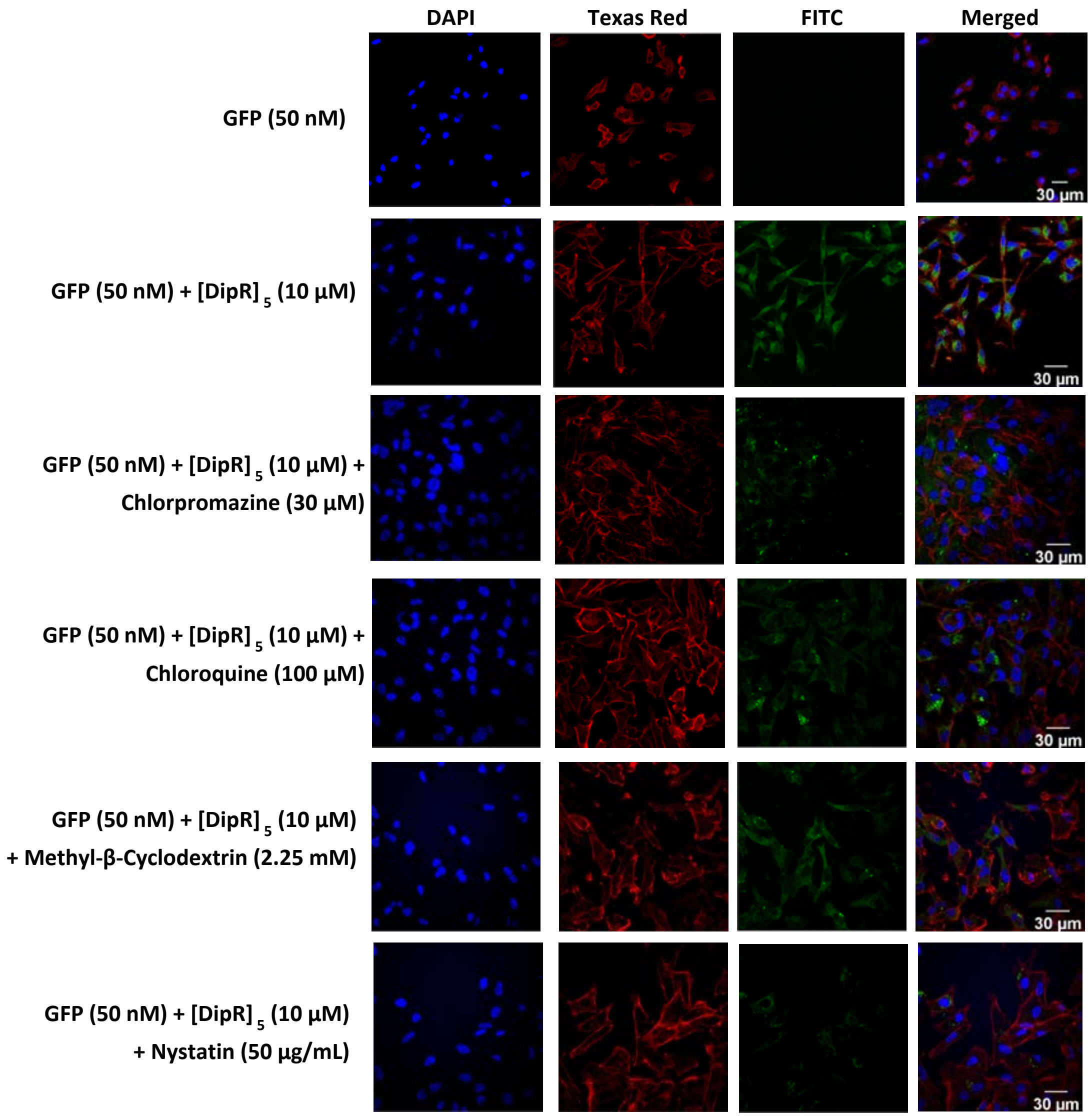

Figure S12. Cellular uptake study of GFP (50 nM) with $[DipR]_5$ in the presence of endocytosis inhibitors in MDA-MB-231 cells after 3 h incubation. The red, blue, and green channels visualize Texas Red (used to stain the cellular cytoskeleton), DAPI (used to stain the nucleus), and GFP, respectively.
